# Cellular Mechanisms of Liver Fibrosis

**DOI:** 10.3389/fphar.2021.671640

**Published:** 2021-05-06

**Authors:** Pragyan Acharya, Komal Chouhan, Sabine Weiskirchen, Ralf Weiskirchen

**Affiliations:** ^1^ Department of Biochemistry, All India Institute of Medical Sciences, New Delhi, India; ^2^ Institute of Molecular Pathobiochemistry, Experimental Gene Therapy and Clinical Chemistry, RWTH University Hospital Aachen, Aachen, Germany

**Keywords:** liver fibrosis, cytokines, chemokines, NASH, therapy, alcohol, cholestasis, drugs

## Abstract

The liver is a central organ in the human body, coordinating several key metabolic roles. The structure of the liver which consists of the distinctive arrangement of hepatocytes, hepatic sinusoids, the hepatic artery, portal vein and the central vein, is critical for its function. Due to its unique position in the human body, the liver interacts with components of circulation targeted for the rest of the body and in the process, it is exposed to a vast array of external agents such as dietary metabolites and compounds absorbed through the intestine, including alcohol and drugs, as well as pathogens. Some of these agents may result in injury to the cellular components of liver leading to the activation of the natural wound healing response of the body or fibrogenesis. Long-term injury to liver cells and consistent activation of the fibrogenic response can lead to liver fibrosis such as that seen in chronic alcoholics or clinically obese individuals. Unidentified fibrosis can evolve into more severe consequences over a period of time such as cirrhosis and hepatocellular carcinoma. It is well recognized now that in addition to external agents, genetic predisposition also plays a role in the development of liver fibrosis. An improved understanding of the cellular pathways of fibrosis can illuminate our understanding of this process, and uncover potential therapeutic targets. Here we summarized recent aspects in the understanding of relevant pathways, cellular and molecular drivers of hepatic fibrosis and discuss how this knowledge impact the therapy of respective disease.

## Introduction

The liver is the largest solid organ in the human body, weighing about 1,200–1,500 g, and comprising about 1/50th of the total body weight in an adult (Dooley et al., 2018). Understanding the complex architecture of the liver is key to understanding liver fibrosis and its consequences.

**FIGURE 1:**
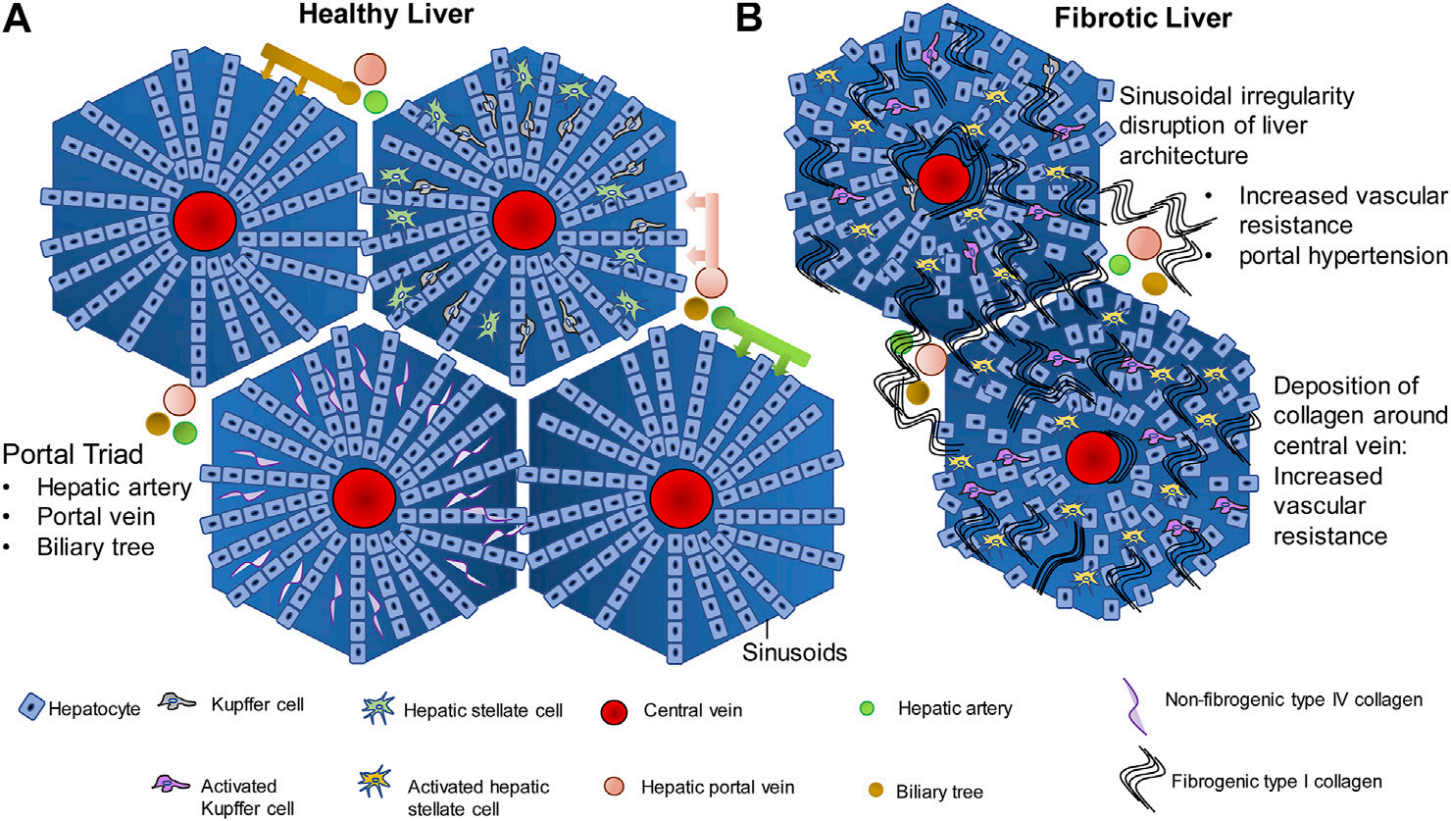
Liver architecture in healthy liver and fibrosis. **(A)** In normal liver, hepatocytes are arranged in rows radiating outwards from the central vein, toward the edge of the lobule. The gaps between the hepatocyte rows are known as sinusoids which are lined with endothelial cells, and contain Kupffer cells, hepatic stellate cells, and contain extracellular material such as the non fibrogenic type IV collagen. Hepatic portal vein, hepatic artery and biliary tree are the three major vessels feeding into the sinusoids and the exchange of blood gases, nutrients and other signaling molecules occurs in the sinusoids. **(B)** Injury to hepatocytes due to any of several causes such as alcohol, drug, genetic predisposition, etc., activates the wound healing fibrogenic response. Chronic injury to the hepatocytes and chronic activation of the fibrogenic pathway in the liver leads to synthesis of fibrogenic type I collagen by the Hepatic stellate cells and its deposition within the sinusoids. Deposition around the central vein and around the portal vein leads to increase in vascular resistance and portal hypertension. Compensatory mechanisms such as esophageal varices and ascites follow.

The liver has two major sources of blood supply, namely (i) the portal vein and (ii) the hepatic artery. The portal vein brings venous blood from the intestines and spleen to the liver. The hepatic artery brings arterial blood to the liver from the celiac axis. The liver is encapsulated by the Glisson’s capsule which is mainly composed of connective tissue (Junqueira and Carneiro, 2002). Within the Glisson’s capsule, the liver is divided into polygonal sections called lobules which are also separated by connective tissue. Each lobule has a characteristic arrangement which is disturbed during liver fibrosis and is completely damaged during cirrhosis (Figure 1A) (Sasse et al., 1992). Since liver function is so intricately linked to this arrangement, hepatic function is completely disrupted during cirrhosis leading to complications. The liver lobule, which is roughly hexagonal, harbors the hepatic central vein at its center (Sasse et al., 1992; Junquiera and Carneiro 2002). Hepatocytes are the most abundant cell type in the liver, constituting about 60% of the total cell number and 80% of liver cell volume. Hepatocytes perform the major roles of the liver such as detoxification of xenobiotics, urea cycle and the synthesis of plasma proteins (Zhou et al., 2016). Hepatocytes are arranged in straight lines radiating out from the central vein toward the edge of the lobule. The space between the radially arranged files of hepatocytes is commonly termed the sinusoids. Bile duct, lymphatics, neurons, as well as the branches of hepatic artery and portal vein line the periphery of the lobules and feed into the liver sinusoids. The portal vein and hepatic artery branch into the liver sinusoids, toward the central vein. Sinusoids are lined with fenestrated endothelial cells, and harbor immune cells such as Kupffer cells, hepatic stellate cells (HSCs) and hepatic natural killer cells (NK cells). These are known as the non-parenchymal cells of the liver. The space between the periphery of the hepatocyte lining and the endothelial cells is known as the space of Disse. The space of Disse is where the exchange of nutrients and other molecules occurs between the hepatocytes and blood flowing through the blood capillaries from the portal vein and the hepatic artery (Sasse et al., 1992). Interactions between the parenchymal and non-parenchymal cells in this carefully preserved architecture are central to efficient functioning of the liver.

Fibrogenesis is a normal wound healing response to tissue injury. All hepatocellular injuries activate the fibrogenic pathways. Once these pathways are activated, fibrogenic components of the extracellular matrix (ECM) are secreted into the space of Disse in order to encapsulate and isolate the damaged portion of the tissue for repair (Bataller and Brenner, 2005). During the encapsulation, there is an infiltration of immune cells that clear cellular debris and initiate tissue repair. The transition from a normal liver to fibrotic liver involves activation and modulation of complex signaling pathways, cell-cell communication between the hepatocytes and non-parenchymal cells, immune system, tissue repair pathways and the extracellular space. In the normal liver, the ECM present in the space of Disse is made up of glycoproteins like fibronectin and laminin, type IV collagen (non-fibrogenic) and proteoglycans such as heparan sulfate (Figure 1A). These components form a lattice-like matrix, which are essential for providing both mechanical support as well as molecular signals for the proper arrangement and functioning of liver cells. When there is hepatic injury, the composition and density of the ECM changes. There is almost a 6–8 fold increase in the production of ECM components. Non-fibrogenic type IV collagen is replaced by fibrogenic type I and II collagen (Figure 1B). There is additional secretion of fibronectin, hyaluronic acid and α-smooth muscle actin into the ECM. In addition, endothelial cell fenestrations as well as microvilli on the hepatocyte basal membrane are lost thereby compromising exchange of nutrients and metabolites as well as other signaling molecules between the circulation and hepatocytes. While the response to tissue injury is a rapid process and fibrogenesis is intended at promoting wound healing, repeated injury and activation of the fibrogenic pathways result in a chronic activation of fibrogenesis (Iredale et al., 2013). This leads to an increased synthesis and decreased degradation of type I collagen over a period of time. This results in deposition of type I collagen in the ECM surrounding the lobules which is the hallmark of fibrosis. Large amounts of type I collagen deposition around the lobules and within the sinusoidal space disrupts the radial arrangement of the hepatocytes, interfering in the flow of nutrients and signaling molecules from the blood through the sinusoids to the hepatocytes, disrupting hepatocyte function. The deposition of collagen around the lobules causes major structural changes in the liver thereby disrupting liver function as well (Iwakiri, 2014). Accumulation of type I collagen fibers leads to mechanical rigidity in the ECM which puts pressure on the blood vessels flowing through the liver. This leads to intrahepatic vasoconstriction and vascular resistance (Pinzani and Vizzuti, 2005). Therefore, in a major vein like the hepatic portal vein, this leads to portal hypertension, which is a major clinical concern in liver fibrosis. The unchecked development of portal hypertension has two major consequences: (i) development of collateral blood vessels from the systemic and splanchnic circulation and, (ii) vasodilation of the hepatic artery (Bosch, 2007). This further increases blood flow into the hepatic portal vein leading to further portal hypertension. As a compensatory mechanism to relieve pressure, submucosal veins present at the lower portion of the esophagus dilate leading to esophageal varices that have the potential to rupture and can be fatal. Fluid begins accumulating within the peritoneal cavity, leading to ascites-the hallmark of advanced decompensated cirrhosis (Bolognesi, et al., 2014; Perri, 2013). These physiological processes are summarized in Figure 1B.

## Drivers of Liver Fibrosis

### Genetic Disorders

Several genetic diseases predispose the liver to fibrosis (Scorza et al., 2014). In all of these diseases, the initiation of fibrosis begins with tissue injury due to a consequence of the genetic defect followed by a fibrogenic wound healing response, as discussed above. Genetic causes for liver fibrosis have come into light due to the advancements in molecular genetic and imaging techniques. Several genetic polymorphisms summarized in Table 1, have been implicated in the occurrence of liver fibrosis, leading to cirrhosis (Pinzani and Vizzutti, 2005). Most of these mutations affect many different cell types but predispose the individual to liver fibrosis and in some cases, liver cirrhosis (Scorza et al., 2014). Many of the genes listed in Table 1, such as, *ABCB4*, *ALDOB*, *GBE1*, *FAH*, *ASL*, *SLC25A13*, and *SERPINA1* are highly expressed in the liver and therefore, mutations in these genes, the liver is the organ which is most affected. Most genetic disorders that lead to cirrhosis manifest in childhood and are a leading cause of pediatric liver cirrhosis, apart from childhood obesity (Pinto et al., 2015). In addition to the genetic mutations that predispose individuals to hepatic fibrosis that appear in childhood, mutations of the *PNPLA3* gene have been described as a major predisposing factor in non-alcoholic fatty liver disease (NAFLD) (Anstee et al., 2020). *PNPLA3* encodes for Patatin-like phospholipase domain-containing protein 3 or adiponutrin and is abundantly expressed in hepatocytes, adipocytes as well as HSCs (Dong, 2019). The *PNPLA3* I148M variant has been shown to have a positive association with hepatic fat content (steatosis), NAFLD, non-alcoholic steatohepatitis (NASH) as well as hepatocellular carcinoma (Dong, 2019). The global prevalence of NAFLD is about 25% and in obese individuals or in the presence of type 2 diabetes mellitus, it increases to about 60% (Younossi et al., 2016). Therefore, *PNPLA3* gene is a strong predisposing genetic factor for hepatic fibrosis. Although the PNPLA3 protein has been shown to have triacylglycerol lipase and acylglycerol transacylase enzymatic activities, its exact role in hepatocytes have been controversial (Jenkins et al., 2004; Dong, 2019). Other studies have demonstrated a retinyl esterase activity for PNPLA3 (Pirazzi et al., 2014). HSCs are reservoirs for retinoic acid, which activate the retinoic acid receptor (RAR) mediated transcription which keeps fibrogenesis under control (Hellemans, et al., 1999; Hellemans et al., 2004; Wang et al., 2002). Mutations in the *PNPLA3* gene that alter the retinyl esterase activity therefore, decrease the level of retinoic acid in the HSCs and therefore reduce the RAR mediated control of fibrogenesis in HSCs (Bruschi et al., 2017). However, it is now recognized that PNPLA3 has pleiotropic roles in the hepatocyte that are still under investigation such as in hepatocyte lipid droplet homeostasis, HSC quiescence and proliferation regulation (Dong, 2019). As several roles for PNPLA3 are suggested, PNPLA3 might be a good therapeutic target to control NAFLD related fibrosis and disease progression.

**TABLE 1:** Genetic causes predisposing the liver to fibrosis.

Disease	Gene	Gene function	Cause of tissue injury	Clinical presentation with liver involvement
Wilson's Disease	*ATP7B*	Copper transport	Intra-hepatic Cu^2+^ accumulation	Variable presentation. Can be asymptomatic or accompanied by fibrosis, acute hepatitis, end stage liver disease
Progressive familial intrahepatic cholestasis type 3	*ABCB4*	Biliary phospholipid secretion	Accumulation of phospholipids and other xenobiotics; impairment of bile formation	Manifests in early childhood, jaundice, splenomegaly, portal hypertension and physical and mental retardation
Hereditary fructose intolerance	*ALDOB*	Converts fructose into trioses for entry into glycolysis and gluconeogenesis	Accumulation of fructose 1 phosphate and depletion of inorganic phosphate levels, inhibition of glycogenolysis, accumulation of high levels of fructose can be hepatotoxic	Hereditary fructose intolerance, hepatotoxicity, liver dysfunction progressing to cirrhosis
Glycogen storage disease type IV	*GBE1*	Glycogen branching enzyme	Accumulation of unbranched glycogen causing hepatotoxicity	Variable presentation. Hepatic classical presentation includes liver dysfunction progressing to cirrhosis, failure to thrive by 5 years of age. The non-progressive hepatic subtype present with hepatomegaly, liver dysfunction, myopathy, and hypotonia; but likely to survive without further progression to cirrhosis
Tyrosinemia type I	*FAH*	Last step in tyrosine catabolism	Accumulation of fumarylacetoacetate and tyrosine in the hepatocytes and oxidative damage to cells	Presentation as liver or renal failure; in early infancy; liver related symptoms are hypoalbunimea, lowering of synthetic functions of the liver, leading to steatosis, cirrhosis and HCC
Hemochromatosis	*HFE*	Interactions with the transferrin receptor and iron uptake	Intra-hepatic iron overload	Presentation as liver cirrhosis
Argininosuccinate lyase deficiency	*ASL*	Urea cycle enzyme that cleaves argininosuccinate into arginine and succinate	Accumulation of urea cycle intermediates, especially ammonia	Two forms:-Early onset in infancy associated with hyperammonimea and vomiting, failure to thrive, or late onset associated with hyperammonimea episodes, cirrhosis and neurological symptoms
Citrin deficiency	*SLC25A13*	Calcium binding mitochondrial carrier protein Aralar2 (exchange of cytoplasmic glutamate with mitochondrial aspartate across the inner mitochondrial membrane)	Citrullinemia and ammonia accumulation	Neonatal intrahepatic cholestasis: impaired bile flow, fibrosis, cirrhosis; late onset citrullinemia 2: neuropsychiatric symptoms
Cholesteryl ester storage disease	*LIPA*	Lysosomal acid lipase (LAL) catalyses the intracellular hydrolysis of triacylglycerols and cholesteryl ester	Intracellular accumulation of cholesteryl esters, triglycerides in the lysosomal compartment of hepatocytes	Early onset: hepatomegaly, splenomegaly and altered serum transaminases
α1 antitrypsin deficiency	*SERPINA1*	Inhibitor of various proteases including trypsin and therefore, protects cells from inflammatory proteases such as from neutrophils	Accumulation of mutant poly-AAT fibers leading to hepatotoxicity	Variable clinical severity ranging from chronic hepatitis and cirrhosis to fulminant liver failure
Cystic fibrosis	*CFTR*	Membrane chloride channel; expressed on the cholangiocytes	Pathogenesis unknown	Age of onset is late: elevation of serum liver enzymes, hepatic steatosis, focal biliary cirrhosis, multilobular biliary cirrhosis, neonatal cholestasis, cholelithiasis, cholecystitis and micro-gallbladder
Alström syndrome	*ALMS1*	Centrosome and basal body associated protein: microtubule organization	Pathogenesis unknown: likely to be involved in cellular Ca^2+^ signaling	Multiple organ dysfunction: liver involvement can range from steatohepatitis to portal hypertension and cirrhosis and can cause hepatic encephalopathy and life-threatening esophageal varices
Congenital hepatic fibrosis	Cryptogenic causes	NA	NA	Multiple organ fibrosis and dysfunction: Can present as the following in case of liver involvement: (i) portal hypertension (most common and more severe in the presence of portal vein abnormality), (ii) cholangitis with cholestasis and recurrent cholangitis, (iii) both portal hypertension and cholangitic symptoms; and (iv) latency that appears at a late age with hard hepatomegaly
Non-alcoholic fatty liver disease (NAFLD)	*PNPLA3*	Pleiotropic role with triglyceride lipase and retinyl esterase activity	Accumulation of triglycerides, impaired retinoic acid receptor signaling and activation of HSC fibrogenic pathway	Hepatic steatosis, fibrosis, cirrhosis, hepatocellular carcinoma

### Alcohol

**FIGURE 2:**
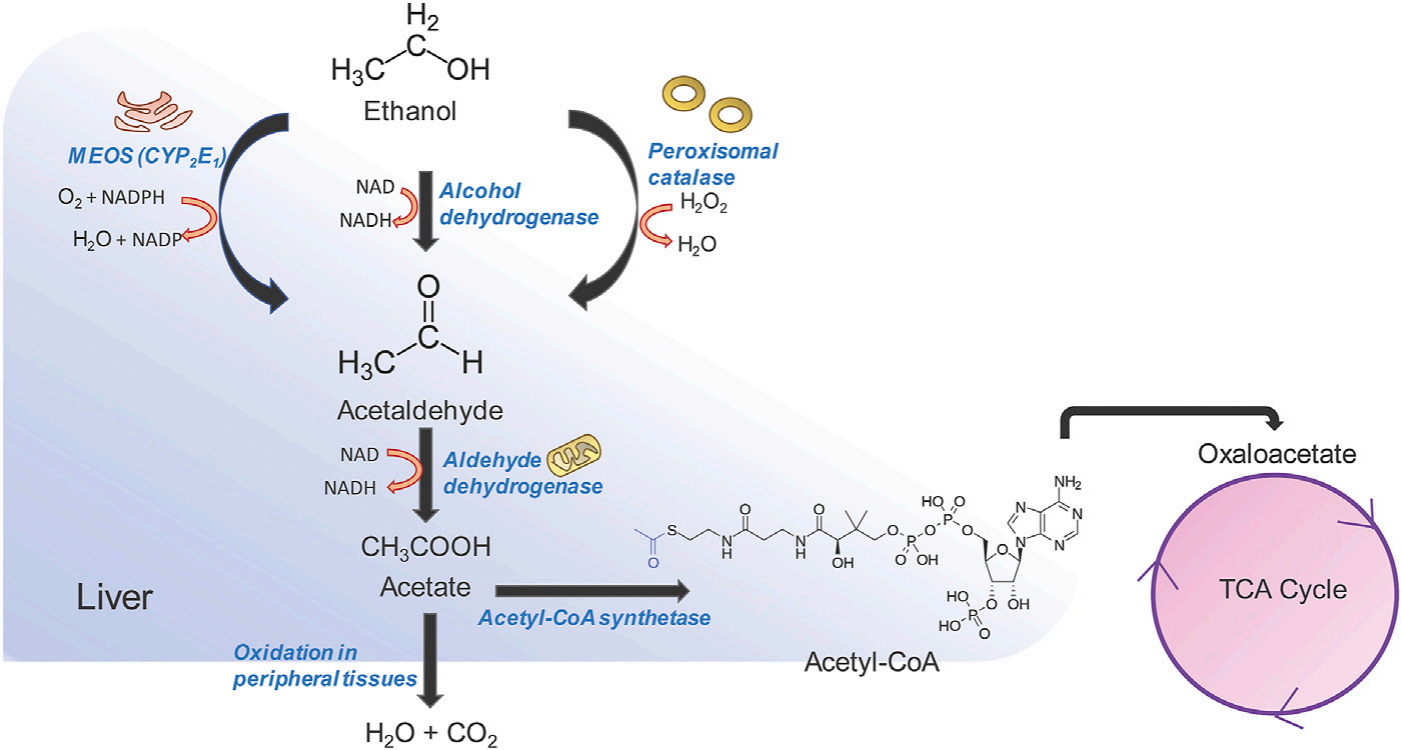
Alcohol metabolism in the liver. Three pathways are involved in alcohol metabolism and all of them converge on the oxidation of ethanol to acetaldehyde. Acetaldehyde is further converted to acetate by aldehyde dehydrogenase in the mitochondria. Acetate can be rapidly oxidized into CO_2_ and H_2_O by peripheral tissues, or can be diverted to the tri-carboxylic acid (TCA) pathway. The oxidation of ethanol to acetaldehyde by microsomal ethanol oxidation system (MEOS) occurs in the smooth endoplasmic reticulum and changes the NADPH/NADP ratio which in turn influences the regeneration of glutathione thereby increasing cellular oxidative stress. The alcohol dehydrogenase pathway is the major pathway and occurs in the cytosol, generating large amounts of NADH. NADH in turn inhibits TCA cycle enzymes and leads to accumulation of acetyl CoA and increase in ketone body generation and acidosis. NADH also inhibits fatty acid oxidation leading to accumulation of fats and causing “fatty liver.” A combination of the above factors leads to tissue injury and activation of the fibrogenic pathway.

Excessive and continued alcohol intake over large periods of time, i.e., alcohol abuse, can lead to liver fibrosis followed by cirrhosis and liver cancer (Stickel et al., 2017). Alcoholic liver disease (ALD) comprises a spectrum of liver disorders ranging from fatty liver, steatosis, fibrosis with varying degrees of inflammation, cirrhosis. Alcohol abuse contributes to almost 50% of chronic liver disease related deaths globally (Rehm and Shield 2019). While the pathophysiology of alcohol induced cirrhosis is not completely understood, alcohol and its metabolic intermediates such as acetaldehyde are thought to play an important role in it. Alcohol is absorbed from the duodenum and upper jejunum by simple diffusion, reaching peak blood concentration by 20 min post ingestion after which it is quickly redistributed in vascular organs (Koob et al., 2014). Alcohol cannot be stored and needs to undergo obligatory oxidation which occurs predominantly in the liver (Figure 2) (Yang et al., 2019). The first step in alcohol oxidation converts alcohol into acetaldehyde. There are three enzymes in the liver that can carry out this reaction (i) alcohol dehydrogenase (ADH) which catalyzes the bulk of ethanol to acetaldehyde conversion, (ii) the alcohol inducible liver cytochrome P450 CYP2E1 (microsomal ethanol oxidizing system or MEOS) and, (iii) peroxisomal catalase. The ethanol to acetaldehyde conversion by ADH generates NADH (Berg et al., 2002). Oxidation of large amounts of alcohol therefore, leads to the accumulation of NADH, which inhibits lactate to pyruvate conversion and promotes the reverse reaction. Lactate to pyruvate conversion is an important means of entry of lactate into gluconeogenesis. As a result, lactic acidosis and hypoglycemia may occur during excessive alcohol consumption. NADH/NAD^+^ ratio also allosterically regulates fatty acid β-oxidation which breaks down long chain acyl CoA to acetyl CoA for entry into TCA cycle (Berg et al., 2002). Since NADH is a product of fatty acid oxidation, an increase in NADH/NAD^+^ ratio provides an allosteric feedback to the fatty acid β-oxidation pathway thereby decreasing the catabolism of fatty acids and leading to their intracellular accumulation. This leads to “fatty liver.” NADH also inhibits two enzymes of the TCA cycle-isocitrate dehydrogenase and α-ketoglutarate dehydrogenase thereby decreasing the consumption of acetyl CoA by the TCA cycle and leading to increase in intra-hepatic acetyl CoA levels. The accumulation of acetyl CoA, in turn, leads to the increased production and release of ketone bodies exacerbating the acidosis already present in the blood due to increased levels of lactate (McGuire et al., 2006). This is known as alcoholic ketoacidosis, which creates a medical emergency. At very high levels of ethanol consumption, the metabolism of acetate becomes compromised leading to the accumulation of acetaldehyde within the hepatocytes. Acetaldehyde can modify the functional groups of many proteins and enzymes irreversibly forming acetaldehyde adducts which leads to a global dysfunction of hepatocytes and eventually, to cell death (Setshedi et al., 2010). The second major pathway for ethanol metabolism is via the inducible cytochrome P450 CYP1E2, also known as the microsomal ethanol oxidizing system (MEOS) (Lieber, 2004). This is located in the smooth endoplasmic reticulum of hepatocytes. In normal people with average to below average alcohol consumption, MEOS forms a minor pathway for intracellular alcohol metabolism (Lieber, 2004). However, it increases manifold upon chronic alcohol consumption. MEOS catalyzes a redox reaction converting molecular oxygen to water and NADPH to NADP (Figure 2). In the liver, glutathione plays an important role in maintaining the cellular redox status and participates in xenobiotic metabolism (Yuan and Kaplowitz, 2009). NADPH is essential in the regeneration of glutathione. The consumption of cellular NADPH leads to a decrease in regeneration of glutathione thereby leading to oxidative stress. This results in cell death and inflammation leading to alcoholic hepatitis which, in itself can be fatal (Morgan, 2007). Often, these processes occur hand in hand. Cellular depletion of glutathione has an additional consequence. Glutathione is required for the detoxification of several drugs including acetaminophen (van de Straat et al., 1987). In the hepatocytes, acetaminophen is modified to form a cytotoxic metabolite known as N-acetyl-p-benzoquinone imine (NAPQI) via CYP2E1 (van de Straat et al., 1987). Conjugation of NAPQI to glutathione results in an S-glutathione product that detoxifies the molecule and allows safe excretion in the urine. However, depletion of glutathione reserves allows unconjugated NAPQI to prevail in the cells which reacts with DNA and proteins to form adducts, thereby causing cytotoxicity and hepatocyte death (Macherey and Dansette, 2015). Long term alcohol use induces CYP2E1 and therefore facilitates rapid NAPQI formation when the liver encounters acetaminophen. At the same time, chronic alcohol abuse leads to low glutathione reserves. A combination of both these changes makes the liver highly susceptible to acetaminophen induced liver injury as well as injury due to other drugs or metabolites that go through the glutathione detoxification pathway. While drug overuse is, in itself a cause for liver injury, in a background of alcoholic liver disease, it can lead to massive liver damage. Damage to hepatocytes, either due to chronic alcohol abuse, exacerbated by drug use, activates the fibrogenic pathway leading to hepatic fibrosis, cirrhosis and hepatocellular carcinoma.

### Drugs

**FIGURE 3:**
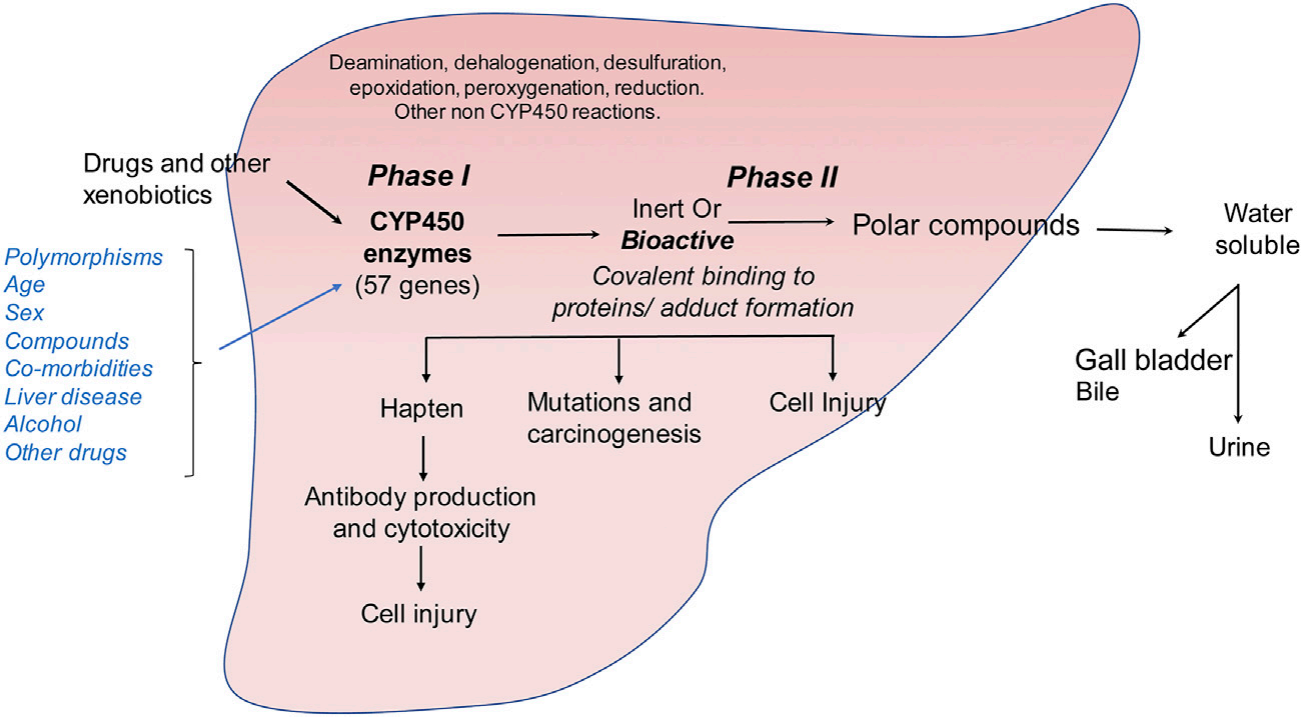
Metabolism of drugs and other xenobiotics in the liver. Drug and xenobiotic metabolism occurs in two phases: (*i*) phase I is catalyzed by the cytochrome P450 family of monooxygenases which metabolize ingested small molecules to form inert or bioactive metabolic intermediates. (*ii*) These intermediates are further catalyzed in phase II reactions to form soluble polar compounds that can be further excreted through urine or bile. Accumulation of bioactive drug or xenobiotic intermediates can lead to the formation of protein or nucleic acid adducts causing autoimmune reaction, carcinogenesis or direct cellular injury.

Drugs induce hepatic fibrosis by causing drug-induced liver injury (DILI) that causes the initiation of fibrogenic tissue repair mechanisms. While the prevalence of DILI is lower as compared to other causes of liver injury, such as alcohol, hepatisis or steatosis, it can lead to life-threatening complications. DILI can be of two types: (i) intrinsic (due to injury caused by a known on-target drug) or (ii) idosyncratic (due to injury caused by an unknown factor and cannot be explained by known pharmacological elements e.g., herbal preparations of unknown compositions) (DiPaola and Fontana, 2018). Among intrinsic causes, acetaminophen induced DILI is the most common. As described above, acetaminophen overload combined with alcohol abuse can exacerbate the liver injury that can occur due to either alcohol or acetaminophen alone. A major function of the liver is detoxification of xenobiotic compounds that enter our circulation either through diet or through intravenous drug usage. Detoxification mechanisms in the liver mainly involve the cytochrome P450 family (*CYP* gene families *CYP1*, *CYP2*, *CYP3*) (McDonnell and Dang, 2013) (Figure 3). Cytochrome P450s are a group of heme proteins that are involved in the initial detoxification reactions of small molecules such as dietary and physiological metabolites, as well as drugs (Zanger and Schwab, 2013; Todorovic Vukotic et al., 2021). The expression of the *CYP* genes is influenced by several factors such as age, sex, promoter polymorphisms, cytokines, xenobiotic compounds and hormones, to name a few (Zanger and Schwab, 2013). Cytochrome P450 mainly carry out a monooxygenation reaction and carry oxidation of drugs/xenobiotic compounds. This can either convert the molecule into an inert or bioactive molecule.

Bioactive compounds can covalently modify intracellular proteins, leading to direct cellular injury, carcinogenesis or production of hapten-protein conjugates that can lead to antibody mediated cytotoxicity (Figure 3). Although the classical view of DILI is that drugs become hepatotoxic as a consequence of or defects in their metabolism, several factors may influence the final outcome of drug intake such as age, gender, comorbidities, intake of alcohol, other drugs or herbal preparations and polymorphisms of the *CYP* genes (Tarantino et al., 2009). The exact mechanism of DILI in specific cases depends on the nature of the molecule and its CYP-transformed metabolites. Drug metabolism can generate free radicals or electrophiles that can be chemically reactive. This can lead to the depletion of reduced glutathione, formation of protein, lipid or nucleic acid adducts and lipid peroxidation. Unless these metabolic intermediates are rapidly neutralized through phase II reactions, they can contribute to cellular stress and injury (Figure 3). They can also lead to modulation of signaling pathways, induce transcription factors, and alter gene expression profiles. In the liver, accumulation of large quantities of reactive drug metabolites can lead to hepatocellular injury, formation of protein adducts that can act as haptens and stimulate production of auto-antibodies or promote cellular transformation. Cellular injury then leads to induction of fibrogenic responses as described above.

### Cholestasis

Cholestasis is emerging as a leading cause for liver injury and fibrosis. Cholestatic liver diseases can occur due to primary biliary cirrhosis and primary sclerosing cholangitis and involve injury to the intra- and extra-hepatic biliary tree (Penz-Österreicher et al., 2011). The pathogenesis of cholestasis is unclear but is believed to have an autoimmune component to it (Karlsen et al., 2017). I primary sclerosing cholangitis (PSC) several strictures appear around the bile ducts and cause bile duct injury. This activates the portal fibroblasts around the bile duct, which then differentiate into collagen secreting myofibroblasts (MFBs) similar to those derived from HSC activation. Recent studies have shown that fibrogenic MFBs have inherent heterogeneity and can be derived from both HSCs and portal fibroblasts (Karlsen et al., 2017). PSC has been shown to be associated with a varied manifestation of other diseases such as inflammatory bowel disease, cholangiocarcinoma, high IgG4 levels, autoimmune hepatitis and colonic neoplasia (Wee et al., 1985; Broomé et al., 1992; Perdigoto et al., 1992; Siqueira et al., 2002; Mendes et al., 2006; Berntsen et al., 2015). Due to its association with autoimmune responses, PSC is thought to involve a genetic predisposition which is activated by an as yet unidentified environmental trigger such as gut dysbiosis (Rossen et al., 2015). Although PSC is traditionally recognized as a rare disease, its incidence is on the rise due to an increase in unknown environmental triggers (Karlsen et al., 2017). Therefore, the pathogenesis of PSC is varied and injury to the bile ducts can occur through multiple pathways. However, the resultant bile duct injury leads to activation of the portal fibroblasts and consequent fibrogenesis.

### Metabolic Disorders: Non-alcoholic Fatty Liver Disease and Non-alcoholic Steatohepatitis

**FIGURE 4:**
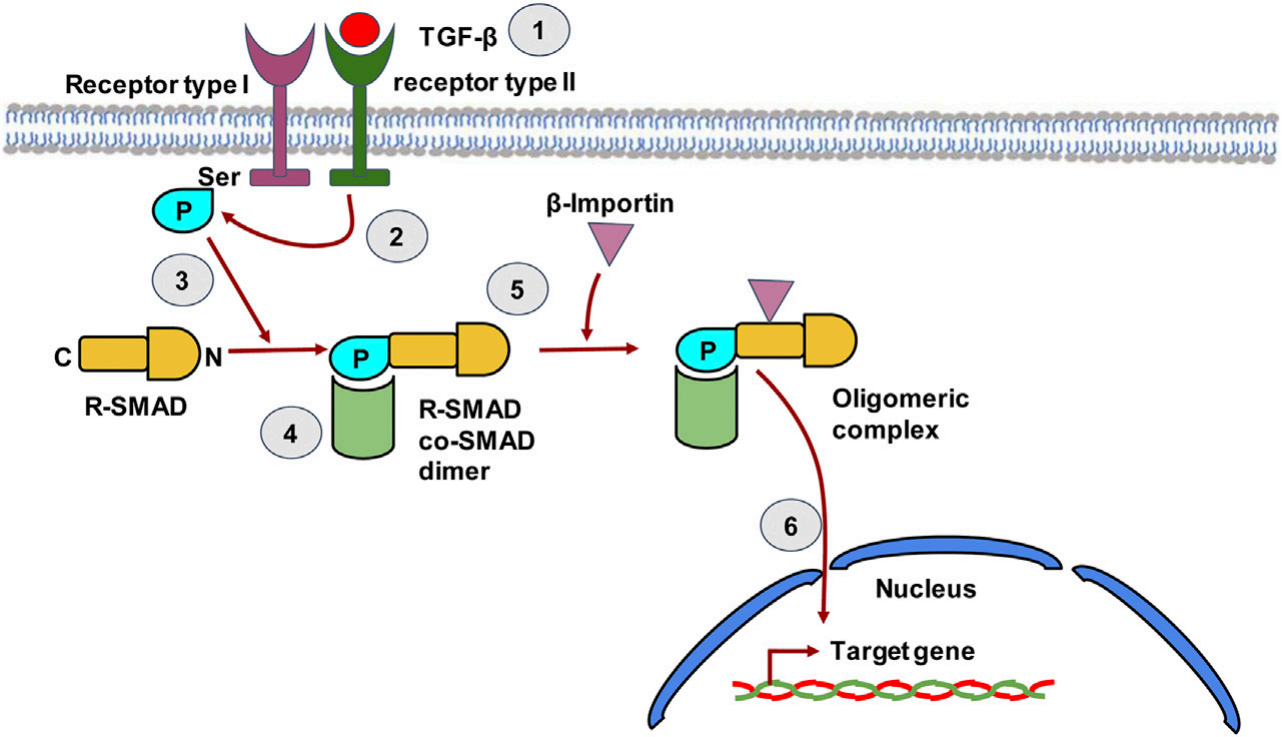
The TGF-β signaling pathway in hepatic stellate cells. TGF-β binds to type II TGF-β receptor leading to receptor dimerization i.e. recruitment of the type I TGF-β receptor. The kinase domain of Type II TGF-β receptor then phosphorylates the Ser residue of type I TGF-β receptor. The phosphorylated receptor now recruits R-SMAD, which binds to receptor through its N-terminal region and gets phosphorylated by the Type II receptor. The C-terminal of R-SMAD has a DNA binding domain (DBD) that can act as a transcription factor. The co-SMAD now binds to R-SMAD and β-Importin binds to the dimer forming an oligomeric complex that guides the R-SMAD and Co-SMAD into the nucleus. The dimer enters the nucleus and the DBD of SMAD now acts as transcription factor that can transcribe target genes.

**FIGURE 5:**
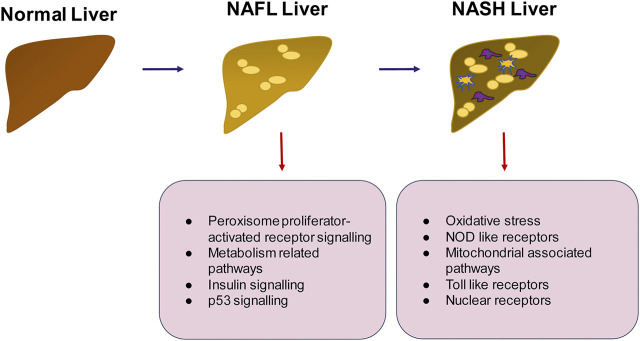
Summary of pathways that may be important in the progression of NAFLD to NASH. The transition from healthy to NAFLD involves the activation of peroxisome proliferator activated receptor signaling, insulin signaling and p53 signaling whereas the switch to NASH involves activation of inflammatory pathways such as TLR and NOD like receptor mediated signaling, generation of intracellular oxidative stress and mitochondrial signaling.

The metabolic syndrome is a group of associated diseases that increase cardiovascular risk factors and are linked with obesity and type 2 diabetes mellitus (Rosselli et al., 2014). Liver manifestations of the metabolic syndrome result in NAFLD (Rosselli et al., 2014). NAFLD is attaining epidemic proportions all over the world. The global prevalence of NAFLD is about 25% and in obese individuals or in the presence of type 2 diabetes mellitus, it increases to about 60% (Younossi et al., 2016). NAFLD is linked to increased risk of hepatic fibrosis, hepatocellular carcinoma and mortality due to cardiovascular disease. The more severe subtype of NAFLD is NASH, which has a global prevalence of about 2–6% and which is associated with severe hepatic inflammation, fibrosis leading to cirrhosis and HCC as well as end stage liver disease (Younossi et al., 2016; Younossi et al., 2019). Recently reported trends in the incidence of NAFLD over time suggest that NAFLD will become the leading cause of end stage liver disease in the decades to come. Emerging data from India, suggests that the national prevalence of NAFLD is about 9–32% in the general population and about 53% in obese individuals (Kalra et al., 2013; Duseja, 2010). Therefore, NAFLD is a global clinical concern. The molecular pathogenesis of NAFLD is complex. However, all pathways in NAFLD converge at the conversion of HSCs into profibrogenic MFBs, through the activation of the TGF-β pathway (Buzzetti et al., 2016) (Figure 4). TGF-β is a pleiotropic cytokine and is involved in various cellular processes like cell proliferation, survival, angiogenesis, differentiation, and the wound healing response (Mantel and Schmidt-Weber, 2011). TGF-β binds to the TGF-β receptor type II, which in turn phosphorylates TGF-β receptor type I thereby recruiting and phosphorylating the intracellular signal transducer proteins belonging to the SMAD superfamily. The SMAD superfamily is composed of intracellular signal transducers that specifically respond to the TGF-β receptor modulation. Phosphorylated SMADs subsequently translocate into the nucleus and control the expression of the TGF-β regulated target genes (Mantel and Schmidt-Weber, 2011) (Figure 4). The activation of HSCs via TGF-β plays a major role in the advanced NAFLD in both experimental animal models, as well as in human liver injury (Yang et al., 2014). In addition to HSC activation, TGF-β signaling followed by SMAD phosphorylation is known to cause hepatocyte death driving progression to NASH (Yang et al., 2017). Hepatocyte death via TGF-β signaling is accompanied by generation of reactive oxygen species as well as lipid accumulation in hepatocytes (Yang et al., 2017). Activation of the TGF-β pathway also leads to HSC differentiation into MFBs leading to formation of fibrillar collagen and exacerbating the combined effects of hepatocyte injury, fibrosis and inflammation, leading to NASH (Yang et al., 2014). While the TGF-β pathway is central to liver fibrogenesis, emerging proteome and transcriptome studies have suggested additional regulatory genes and pathways. These studies have been carried out in animal models of NAFLD or NASH and human liver biopsies obtained from patients. Comparative transcriptomic studies between mouse models of NAFLD and human liver biopsies obtained from NASH patients reveal major differences between human NASH liver transcriptome and mouse NAFLD transcriptomes even at severe stages (Teufel et al., 2016). This suggests major pathophysiological differences between human disease and animal models of the disease and the need to design studies in humanized models of disease or in liver organoid systems (Suppli et al., 2019). A meta-analysis of transcriptomic studies carried out with human liver biopsies suggests the upregulation of several genes within the lipogenesis pathway (Table 2). Interestingly, genes such as *ACACA* (Acetyl carboxylase 1) which catalyzes the synthesis of malonyl CoA from acetyl CoA, the rate limiting step in fatty acid biosynthesis and *ACACB* (Acetyl carboxylase 2) which regulates fatty acid oxidation, are associated with NAFLD liver tissue demonstrating the association of lipogenic functions within the tissue with active disease (Table 2) (Widmer et al., 1996; Locke et al., 2008). In several cases, NAFLD has been shown to be linked to progression toward hepatocellular carcinoma. Recent studies have led to the understanding that the evolution of NAFLD to NASH and HCC is multifactorial and involves the innate immune system to a great extent (Chen et al., 2019). Lipid accumulation and mitochondrial dysfunction have been identified as critical components of the pathways leading to NAFLD (Margini and Dufour, 2016). Many new genes and pathways have been implicated at every stage of NAFLD to NASH to HCC progression (Figure 5). Regulation in PPAR-γ, Insulin and p53-mediated signaling have been implicated in NAFLD development, whereas signatures of inflammatory signaling such as Toll-like receptor (TLR) and Nucleotide-binding, oligomerization domain (NOD) protein signaling pathways, in addition to pathways reflecting mitochondrial dysfunction characterize NASH (Figure 5) (Ryaboshapkina and Hammar, 2017).

**TABLE 2:** Summary of pathways from transcriptomics analyses implicated in NAFLD

Gene		Function/remarks	References
***LEP***	Leptin	Anti-steatotic, but also a proinflammatory and profibrogenic action	Polyzos et al. (2015)
***PEMT***	Phosphatidylethanolamine *N*-methyltransferase	Governs the secretion of hepatic triglycerides in the form of very low-density lipoprotein	Tan et al. (2016)
***PPAR-γ2***	Peroxisome proliferator activated receptor gamma	Pparγ2 is expressed in the liver, specifically in hepatocytes, and its expression level positively correlates with fat accumulation induced by pathological conditions such as obesity and diabetes	Lee et al. (2018)
***TNF-α***	Tumor necrosis factor	Tumor necrosis factor (TNF)-α is associated with insulin resistance and systemic inflammatory responses	Seo et al. (2013)
***PNPLA3***	Patatin like phospholipase domain containing 3	Polymorphisms in *PNPLA3* have been linked to obesity and insulin sensitivity	Chen et al. (2010)
***CD14***	CD14 molecule	Upregulation of *CD14* in liver cells show increased sensitivity to LPS, changes in *CD14* expression could represent a mechanism regulating liver sensitivity to LPS toxicity	Satoh et al. (2013)
***ACACA***	Acetyl-coa carboxylase α	Low level is correlated with long time survival	Liu et al, (2020)
***ACACB***	Acetyl-coa carboxylase β	Involved in insulin signaling pathway and adipokine metabolic pathway	Li et al. (2019)
***ASPG***	Asparaginase	The bacterial enzyme L-Asparaginase is a common cause of anti-neoplastic-induced liver injury with occurrence of jaundice and marked steatosis	Kamal et al. (2019)
***CCS***	Copper chaperone for superoxide dismutase	*CCS* expression is regulated by copper by modulating its degradation by the 26S proteosome	Bertinato and L’Abbé (2003)
***CHEK1***	Checkpoint kinase 1	This kinase is necessary to preserve genome integrity	Zhang and Hunter (2014)
***HDAC9***	Histone deacetylase 9	Downregulation of *HDAC9* decrease TGF-β1-induced fibrogenic gene expression in hepatic stellate cells	Yang et al. (2017)
***NADSYN1***	NAD synthetase 1	Reduced NAD concentrations contribute to the dysmetabolic imbalance and consequently to the pathogenesis of NAFLD	Guarino and Dufour (2019)
***NHP2L1***	Small nuclear ribonucleoprotein 13	This genes encodes a protein of the spliceosome complex	Liu et al. (2020)
***OAS3***	2′-5′-oligoadenylate synthetase 3	OAS3 is an interferon-induced aniviral enzyme	Zhang and Yu (2020)
***PCNA***	Proliferating cell nuclear antigen	*PCNA* encodes the protein which is found in the nucleus and is a cofactor of DNA polymerase delta and involved in the RAD6-dependent DNA repair pathway in response to DNA damage	Xing et al. (2018)
***RPL10L***	Ribosomal protein L10 like	The encoded protein shares sequence similarity with ribosomal protein L10	Liu et al. (2020)
***RSL24D1***	Ribosomal L24 domain containing 1	The encoded protein is involved in involved in the biogenesis of the early pre-60S ribonucleoparticle	Xie et al. (2020)
***SRC***	*SRC* proto-oncogene, non-receptor tyrosine kinase	*SRC* is a proto-oncogene encoding a non-receptor tyrosine kinase	Amanatidou and Dedoussis (2021)
***TOP2A***	DNA topoisomerase II alpha	Regulates the topologic states of DNA and controls tumor cell response	Wong et al. (2009)
***TP53***	Tumor protein p53	Induces apoptosis but the association between p53 and NAFLD remains controversial, P53 plays an essential role in the pathogenesis of NAFLD, whereas others have indicated that suppression of p53 activation aggravates liver steatosis	Yan et al. (2018)
***TWISTNB***	RNA polymerase I subunit F	This gene (i.e. TWIST Neighbor) is ubiquitous expressed in all tissues	Liu et al. (2020)
***UMPS***	Uridine monophosphate synthetase	Lack of this gene results in reduced cell membrane stability	Wortmann and Mayr (2019)
***HORMAD2***	HORMA domain containing 2	Decreases with advancing fibrosis	Liu et al. (2020)
***LINC01554***	Long intergenic non-protein coding RNA 1554	*LINC01554*, one kind of lncRNA, has been found specifically enriched in liver tissue and have strong association with pathogenesis and clinical evaluation of HCC	Ding et al. (2020)

There are only a limited number of proteomics studies in human NAFLD. A comparative quantitative proteomics study between NAFLD and Metabolic Healthy Obese (MHO) individuals was carried out using liver tissue obtained during surgery (Yuan et al., 2020). This study demonstrated the relevance of PPAR signaling, ECM-receptor interaction and oxidative phosphorylation in resisting NAFLD. Proteins upregulated in NAFLD were involved in organization of the ECM, and proteins downregulated in NAFLD were involved in redox processes. A schematic of pathways relevant in NAFLD progression, as gleaned from various “omics” approaches is summarized in Figure 5.

### Viral Hepatitis

In older children, autoimmune hepatitis and viral hepatitis are the leading causes of liver fibrosis followed by cirrhosis. Viral hepatitis can be caused by any one of the five viruses: Hepatitis A, B, C, D, and E of which A and E are usually acute, while B, C, and D are chronic (Zuckerman 1996). All hepatitis viruses are infectious, while alcohol, other toxins and autoimmune mediated hepatitis are usually non-infectious. HBV and HCV lead to hepatic inflammation (Gutierrez-Reyes et al., 2007). Several viral components are known to induce cellular damage in hepatocytes and liver constituents. For instance, the HCV core protein in chronic infections is known to interact with the TNF-α receptors (TNFRSF1A) which subsequently induces a pro-apoptotic signal in hepatocytes (Zhu et al., 1998). Polymorphisms in *TNFRSF1A* have been shown to be associated with HCV outcomes (Yue et al., 2021). The HCV core protein is also known to interact with ApoA1and ApoA2, thereby interfering with the assembly and secretion of very low density lipoprotein (VLDL), thus cause the accumulation of triglycerides in the liver through the interaction of both viral and metabolic factors and subsequent cell death (Gutierrez-Reyes et al., 2007). Furthermore, the viral core protein as well as the HCV non-structural protein 5A (NS5A) are known to cause mitochondrial ROS production and cellular stress leading to cell death (Bataller et al., 2004). Interestingly, HCV and NAFLD can co-exist and have been shown to have a more rapid disease progression than either disease alone (Patel and Harrison, 2012; Dyson et al., 2014). About 50% of HCV patients have steatosis with significant fibrosis and the HCV genotype 3 is mainly associated with the steatosis, however the exact mechanism leading to steatosis in HCV patients is not fully elucidated.

The association of hepatitis B virus (HBV) infection with NAFLD however, appears to be controversial. Some studies suggest that HBV infection is protective against steatosis, insulin resistance and metabolic syndrome (Morales et al., 2017; Xiong et al., 2017) while others suggest that chronic HBV infections can co-exist with NAFLD and can actively worsen the disease (Zhang et al., 2020). The presence of Hepatitis B protein X (HBx) in the cells has been shown to increase the production of reactive oxygen species (ROS) increasing the formation of lipids in the cells and therefore HBx could be a risk factor for the development of NAFLD (Wang et al., 2019). Therefore, an alternative mechanism by which viral hepatitis can induce fibrosis is through their ability to cause NAFLD.

### Parasitic Infections

The liver is capable of hosting a wide range of parasites which vary in host cell requirement (extra or intracellular), sizes (unicellular to multicellular) and potential harm to the host cells or organs (Dunn, 2011). Parasites which have co-evolved with humans through centuries, such as the malaria parasites cause minimal injury to the host liver and move on to the blood with ease (Acharya et al., 2017). However, some parasites can cause injury to the cells of the liver and trigger the activation of the fibrogenic pathway. Some of these parasites are discussed below:

Leishmania is an intracellular protozoan parasite that infects the reticuloendothelial system (RES) in the body, i.e., circulating monocytes as well as tissue-resident macrophages (Magill et al., 1993). Leishmaniasis is transmitted by the bite of infected sandflies (Dunn, 2011). Visceral leishmaniasis (kala-azar) involves the RES infection of the visceral organs like the liver, spleen, bone marrow and other lymph nodes. Kupffer cells, the tissue resident macrophages of the liver, take up the amastigote stage of Leishmania from circulating infected reticuloendothelial cells. The parasite then replicates within the macrophages and activates the host inflammatory and Th1 and Th17 mediated adaptive immune responses in immunocompetent individuals (Pitta et al., 2009). Leishmaniasis is typically associated with increased liver fibrosis (Melo et al., 2009). Leishmania parasites have been shown to use host ECM components such as fibronectin and laminin to access Kupffer cells for infection (Wyler et al., 1985; Wyler, 1987; Vannier-Santos et al., 1992; Figueira et al., 2015). Visceral leishmaniasis has been frequently studied in dogs as a model system. These studies suggest that dogs infected with Leishmania have a significantly higher level of collagen and fibronectin deposition (Melo et al., 2009). Intra-lobular collagen deposition, appearance of MFBs and effacement of the space of Disse are characteristic of overt Leishmania infection in slightly or severely immunocompromized individuals (Dunn, 2011). Leishmania associated fibrosis is completely reversible once the parasitic infection has been treated. However, since overt disease and severe fibrosis usually occurs in immunocompromized individuals such as those infected with HIV, relapses typically occur once treatment ceases (Dunn, 2011).

Schistosomiasis is caused by Schistosoma species which are a group of blood flukes belonging to the trematode or flatworm family (Andrade, 2009). It is prevalent mainly in the tropical and sub-tropical regions of the world. Schistosoma use freshwater snails as intermediate hosts, which release eggs into water bodies which then come into contact with humans and infect them (WHO. World Health Organization, 2021). Schistosomiasis can be intestinal (wherein the liver is involved) or urogenital. Intestinal schistosomiasis can be caused by many different species such as *Schistosoma mansoni* (found in Africa, Middle East, Caribbean, Brazil, Venezuela and Suriname), *S. japonicum* (found in China, Indonesia and the Philippines), *S. mekongi* (Cambodia, and the Lao People’s Democratic Republic), *S. guineensis* and *S. intercalatum* (found in the rain forests of central Africa). Urogenital infection is caused by *S. hematobium* (found in Africa, the Middle East and Corsica in France) (WHO. World Health Organization, 2021).

*Schistosoma mansoni* are associated with liver fibrosis (Andrade, 2009). Schistosome eggs are carried to the liver by the portal vein and stop in the pre-sinusoidal vessels (Andrade, 2004). The development of severe schistosomiasis is thought to have two components- (a) a major determinant is the high worm load and, (b) a secondary determinant is thought to be genetic predisposition. At low to moderate worm loads, many patients are asymptomatic and the lesions heal automatically due to the appropriate activation of T-cell mediated host immune responses (Andrade, 2004). A high worm load is also associated with damage to the portal vein and appearance of MFBs and collagen deposition around the portal stem leading to portal fibrosis called pipestem fibrosis (Andrade et al., 1999). Since all infected individuals do not develop severe liver disease, or liver fibrosis, schistosomiasis linked liver fibrosis development is also thought to have a genetic component. A metaanalysis of genetic polymorphisms associated with severe liver disease and fibrosis in schistosomiasis reveals several genetic polymorphisms (Dessein et al., 2020). Several polymorphisms in genes related to the TGF-β pathway were found to be associated with severe fibrosis in schistosomiasis e.g., *TGFBR1*, *TGFBR2*, *ACVRL1*, *SMAD3* and *SMAD9* (Dassein et al., 2020). Polymorphisms in the connective tissue growth factor (CTGF) as well as the IL-22 pathway were also observed. In addition, several associations have been reported between severe hepatic fibrosis during Schistosomiasis and genes encoding for IL-13, TNF-α, MAPKAP1, ST2, IL-10, M1CA, HLADRB1, IL-4, ECP, and IFN-γ, have been reported from various studies (Hirayama et al., 1998; Chevillard et al., 2003; Eriksson et al., 2007; Gong et al., 2012; Silva et al., 2014; Zhu et al., 2014; Long et al., 2015; Oliveira et al., 2015; Long et al., 2017; Silva et al., 2017). These observations suggest that while infectious agents such as schistosoma can drive hepatic fibrosis by mediating tissue damage, genetic predispositions to TGF-β pathway activation or a specific inflammatory response may make the hepatic environment conducive to fibrosis in the presence of an infectious agent.

*Fasciola hepatica*, also known as the liver fluke is also a trematode parasite that infects humans (Machicado et al., 2016). Fascioliasis is a neglected tropical disease. A recent meta-analysis has found an association of Fasciola infections with liver fibrosis, cirrhosis and hepatocellular carcinoma (Machicado et al., 2016). The mechanism of fibrosis development is thought to be due to the activation of HSCs by parasite encoded cathepsins (Marcos et al., 2011). As with schistosoma, worm-load seems to be an important determinant of fibrosis. However, there are a very limited number of studies available on the pathogenesis, molecular epidemiology and prevalence of fascioliasis with liver fibrosis and this area needs further investigation.

### Cryptogenic Causes

Cryptogenic causes of liver fibrosis are cases with unknown causes but it is believed that a high proportion of the cryptogenic liver fibrosis cases could be linked to NAFLD or NASH (Caldwell, 2010; Patel et al., 2020). Other causes could include occult alcohol intake, viral hepatitis, autoimmune hepatitis, biliary disease, vascular disease, celiac disease, mitochondriopathies, systemic lupus erythematosus, Alstrom syndrome, Apolipoprotein B with LDL cholesterol, and genetic disorders such as short telomere syndrome, keratin 18 mutations and glutathione-S-transferase mutations (Caldwell, 2010; Patel et al., 2020).

## Soluble Mediators in Liver Fibrosis

The development of liver fibrosis occurs as a result of interaction between several different cell types including hepatocytes, HSCs, Kupffer cells, as well as infiltrating immune cells. These inter-cellular interactions involve several soluble and secreted mediators which regulate inflammatory pathways, chemotaxis and HSC activation. Some of the known soluble mediators are briefly discussed below.

### Cytokines and Chemokines

Cytokines are regulatory soluble small molecular weight proteins or glycoproteins released by several cells and mediate interaction, communication between different cell types. Cytokines play an important role in the progress of liver fibrosis (Xu et al., 2012). In liver they mediate the interactions of the various cell types and contribute to either the production of proinflammatory or hepatoprotective responses (Kong et al., 2012). Cells of the immune system such as Kupffer cells and neutrophils produce many cytokines and chemokines that can affect the gene expression, proliferation, contractility and activation of HSCs. The interaction between HSCs and immune cells are bidirectional, i.e., while immune cells produce cytokines to activate HSCs. HSCs also regulate immune cell chemotaxis and response by secreting soluble mediators themselves (Weiskirchen, 2016). For instance, the pro-inflammatory cytokines TGF-α increases HSC proliferation, TGF-β inhibits HSC apoptosis and promotes ECM remodeling leading to a pro-fibrogenic phenotype, TNF-α inhibits HSC apoptosis and induces chemokines and ICAM-1 in HSCs (Maher, 2001). IL-4 in concert with MMP-2 and ROS increase ECM synthesis and fibrosis. At the same time, anti-fibrogenic cytokines are also released from immune cells that can control the pro-fibrogenic HSC activation, such as IL-10, IFN-α, IFN-γ. A balance of these factors results in a net pro-or anti-fibrogenic effects on HSCs. The HSCs also secretes several molecules which are instrumental in recruiting immune cells at the site of activation such as M-CSF that causes macrophage proliferation and maintenance, PAF, MIP-2 and CINC/IL-8 which cause neutrophil chemotaxis and MCP-1 which recruits monocytes (Maher, 2001).

While activation of the TGF-β pathway is a central event in the induction of hepatic fibrosis, HSC activation is regulated by other pathways and molecular mechanisms as well, such as the Hippo pathway and autophagy (Tsuchida and Friedman, 2017). The Hippo signaling pathway is an evolutionarily conserved pathway that derives its name from its key player, the protein kinase “Hippo”, which is involved in the regulation of cell and organ size (Saucedo and Edgar, 2007). However, Hippo pathway components such as the transcriptional co-activator Yes-associated protein 1 (YAP1) and the protein kinases macrophage stimulating 1 (MST1) and MST 2 have been shown to be important in initial HSC activation (Manmadhan and Ehmer, 2019). Inhibition or silencing of YAP1, and inactivation of MST1 and MST2 have been shown to have therapeutic effects in mouse models of fibrosis but the human clinical impact of such approaches is presently not known (Manmadhan and Ehmer, 2019).

Chemokines are a subgroup of cytokines that have chemotactic properties. They are synthesized by most liver cells as well as by infiltrating immune cells and their effects depend on their local concentrations at the site of injury (Sahin et al., 2010). Typically, chemokines bind G-protein coupled receptors (GPCRs) and induce signaling in target cells (Bonecchi et al., 2009). Stellate cells express several chemokines as well as chemokine receptors. HSCs have been shown to secrete CCL2, CCL3, CCL5, CXCL1, CXCL8, CXCL9 and CXCL10 (Holt et al., 2009; Wasmuth et al., 2009; Zaldivar et al., 2010; Marra and Tacke, 2014). Portal fibroblasts which are involved in cholestastis-associated fibrosis are also capable of secreting chemokines (Dranoff and Wells, 2010]. Targeting of chemokines and chemokine receptors in experimental models of fibrosis has been shown to control fibrosis and therefore warrants further investigation as a potential therapeutic anti-fibrosis strategy (Sahin et al., 2010).

In addition to these cytokines and chemokines, several miRNA have been recently identified to be involved in the HSC-immune cell cross-talk (Zhangdi et al., 2019).

### Lipid Mediators

Lipid mediators in hepatic fibrosis are mainly studied in the context of NAFLD and NASH (Liangpunsakul and Chalasani, 2019). Several different types of lipid species have been shown to be associated with NAFLD such as saturated free fatty acids (FFA), diacylglycerols, ceramides, lysophosphatidylcholine, eicosanoids and free cholesterol (Feldstein et al., 2003; Caballero et al., 2009; Gorden et al., 2011; Luukkonen et al., 2016). Increased triglyceride accumulation is a hallmark of NAFLD and is associated mainly with hepatic steatosis (Yamaguchi et al., 2007). While triacylglycerol (TAG) accumulation has not been found sufficient for causing insulin resistance, excessive TAG accumulation can increase mechanical pressure on hepatic sinusoids leading to the impairment of hepatic blood flow, and generation of compensatory collateral flow (Wanless and Shiota, 2004). Excessive amounts of free fatty acids can act directly as TLR agonists in the liver or are taken up by the liver, converted into lipotoxic intermediates that activate the JNK, IKK pathway leading to cell injury, inflammation and apoptosis (Yu et al., 2002).

### Extracellular Vesicles

Cellular injury to hepatocytes can lead to many outcomes. In addition to hepatocyte cell death, injured and stressed hepatocytes have been shown to release extracellular vesicles (EV) (Ibrahim et al., 2016; Schattenberg and Lee, 2016). EV are nanovesicles released by almost all cell types (Dooyle et al., 2018). They constitute two major size categories, namely plasma membrane derived microvesicles (50–1,000 nm) and endosome-derived exosomes (30–150 nm in diameter) as defined by the International Society for Extracellular Vesicles” (ISEV) and according to the Minimal Information for Studies of Extracellular Vesicles (MISEV) guidelines of 2014 (Lötvall et al., 2014). In fact, lipid overload has been shown to activate hepatocyte signaling through the death receptor 5 (DR5) followed by release of hepatocyte-derived pro-inflammatory EV containing TNF-α (Cazanave et al., 2011). These EV activated macrophage induced inflammation leading to further cellular injury and the development of NASH in experimental mouse models (Cazanave et al., 2011). Administration of EV isolated from high fat diet (HFD) mice into normal fed mice have been shown to result in exacerbation of hepatic steatosis and accumulation of activated myeloid cells in the liver through the release of chemotactic EV (Ibrahim et al., 2016). In addition to hepatocyte-derived EV, extra-hepatic EV have also been implicated in the progression of NAFLD, NASH and associated fibrosis (Srinivas et al., 2021). Due to their ability to carry signal from one cell type to another, they can activate or modulate target cell responses and are therefore an emerging therapeutic targets in NAFLD and NASH.

### Autophagy and Unfolded Protein Response

Autophagy in response to endoplasmic reticulum (ER) stress has also been recognized as an activator of HSCs. Under normal circumstances, autophagy is an important regulator of hepatic homeostasis (Mallat et al., 2014). While normally, autophagy is believed to have a protective effect on injured hepatocytes, recent studies demonstrate that ER stress signals activate autophagy and a profibrogenic phenotype in HSCs (Mallat et al., 2014). HSC activation is linked to increased flux in autophagy-related metabolic pathways and inhibition of this process can prevent HSC activation (Thoen et al., 2012). Similarly, there is evidence that the accumulation of misfolded or unfolded proteins in the ER triggering a process called unfolded protein response is a critical feature during early activation of profibrogenic cells such as HSCs (Mannaerts et al., 2019), suggesting that the development of interventions targeting the processes of autophagy or unfolded proteins response might be effective in therapy of hepatic fibrosis.

## Cellular Mediators of Hepatic Fibrosis

**FIGURE 6:**
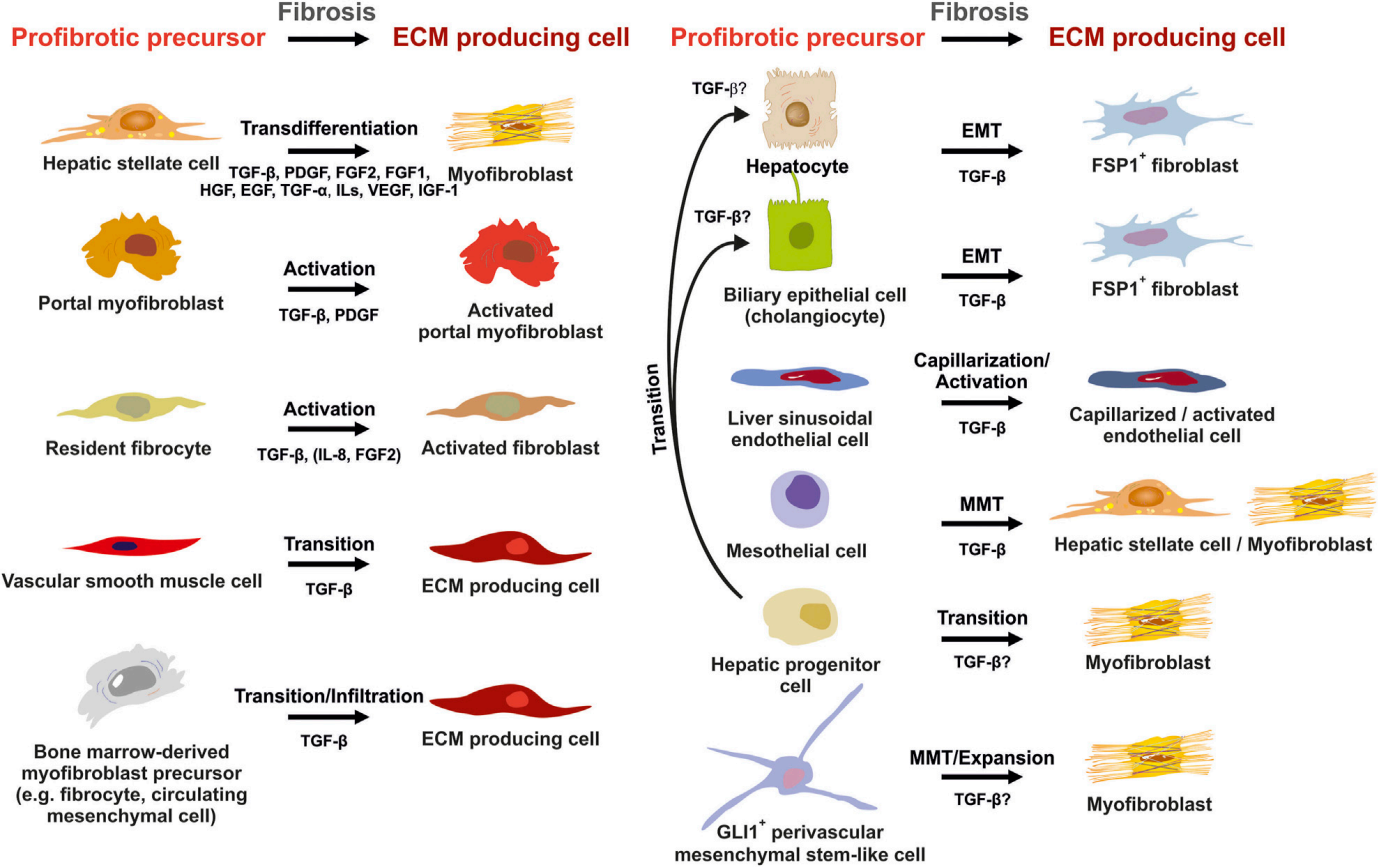
Potential sources of extracellular matrix (ECM) producing cells in liver fibrosis. ECM producing cells during hepatic fibrosis can originate from many sources. Hepatic stellate cells (HSCs) that transdifferenatiate into myofibroblasts (MFBs), activated portal myofibroblasts and activated resident fibroblasts are rich sources of ECM. In addition, several other cell types that become activated, infiltrate the liver, or originate by diverse transition processes are suitable to express large quantities of ECM. Major pathways driving establishment of myofibrogenic features are indicated for each progenitor. Abbreviations used are: ECM, extracellular matrix; EGF, epithelial growth factor; EMT, epithelial-to-mesenchymal transition; FGF1/2, fibroblast growth factor 1/2; GLI1, glioma-associated oncogene homolog 1; HGF, hepatocyte growth factor; IGF-1, insulin growth factor-1; IL, interleukin; MMT, mesothelial-to-mesenchymal transition; PDGF, platelet-derived growth factor; TGF-α/β, transforming growth factor-α/β; VEGF, vascular endothelial growth factor. For details see text.

The hallmark of hepatic fibrosis is the increased expression and deposition of ECM compounds. There are different resident and infiltrating cells that can either be activated or produced by progenitors that transform into a phenotype capable to synthesize ECM. Each of these cell types have specific pro-fibrogenic features and expression potential. Other cells invade the inflamed tissue and acquire a matrix-synthesizing phenotype by reprogramming their cell fate (Figure 6). In most cases, TGF-β regulated pathways contribute to the acquirement of fibrogenic features. However, this might be due to the fact that several cell types were only recently added to the list of profibrogenic progenitors and relevant signaling pathways still need to be defined. In the following we will discuss how the different cells contribute to hepatic fibrosis.

### Hepatic Stellate Cells and Myofibroblasts

**FIGURE 7:**
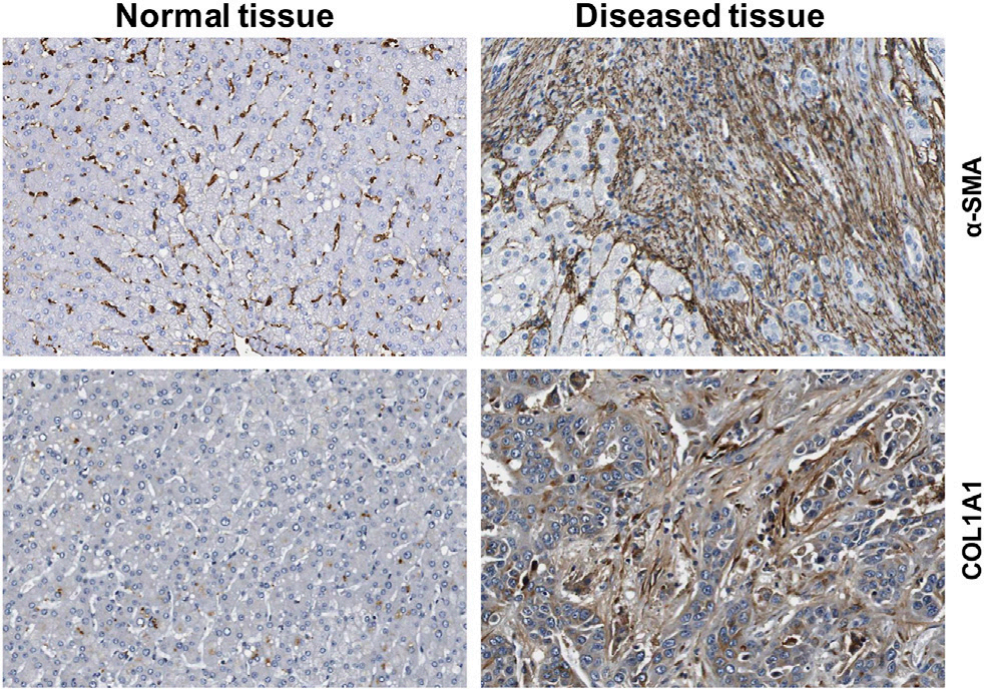
Expression of fibrogenic markers in liver. The figure was compiled using immunohistochemical data from the Human Protein Atlas (www.proteinatlas.org/) (Uhlén et al., 2015). α-smooth muscle actin (α-SMA) and collagen type 1α1 (COL1A1) proteins were stained in normal and diseased livers.

**FIGURE 8:**
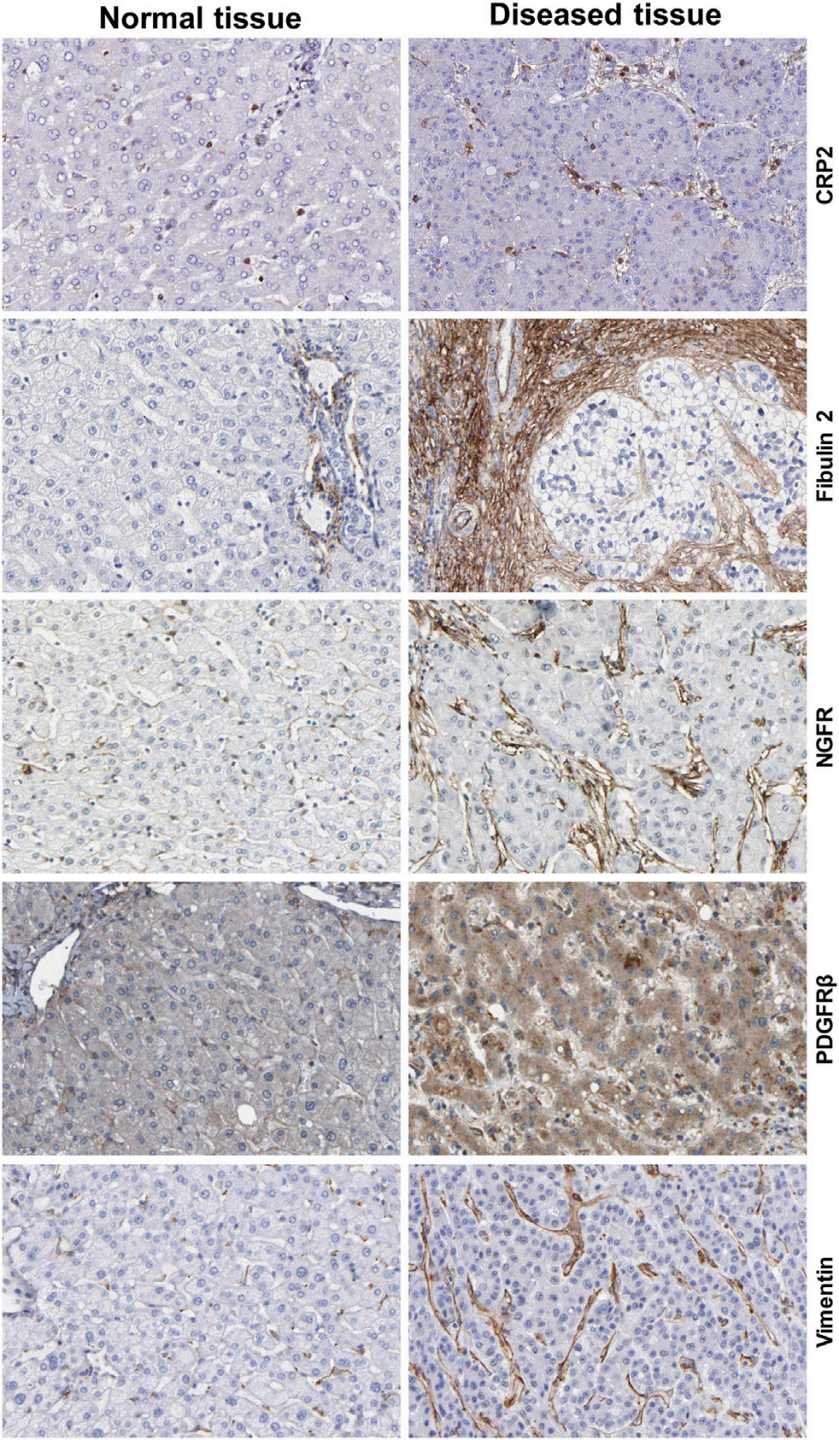
Additional markers of hepatic stellate cells and portal myofibroblasts. The figure was compiled from data deposited from Human Protein Atlas (www.proteinatlas.org/) (Uhlén et al., 2015). Immunohistochemistry of the cysteine and glycine rich protein 2 (CRP2), Fibulin 2, nerve growth factor receptor (NGFR), platelet-derived growth factor-β (PDGFRβ) and Vimentin in normal and diseased liver tissue. Liver damage is associated with increased expression of these profibrogenic markers. Image credit: Human Protein Atlas.

HSCs reside in the perisinusoidal space between hepatocytes and the sinusoids (i.e., the space of Disse). In the normal liver, these cells exhibit a quiescent phenotype with the main known function of storing vitamin A. During chronic hepatic disease, these cells progressively lose their vitamin A, become activated and transdifferentiate into fibrogenic MFBs that are supposed to be the central cellular drivers of hepatic fibrosis in experimental and human liver injury (Tsuchida and Friedman, 2017). In this process, the induction of α-SMA is the most reliable marker indicating cellular activation HSC. Fundamental fate tracing experiments in mice have demonstrated that HSCs are the most important profibrogenic cell type in the liver giving rise to 82–96% of all MFBs in models of toxic, cholestatic and fatty liver disease (Mederacke et al., 2013). HSCs typically express desmin and vimentin, but other markers such as glial fibrillary acidic protein (GFAP), lecithin retinol acyltransferase (LRAT), synemin, platelet-derived growth factor receptor-β (PDGFRβ), p75 neurotrophin receptor peptide (p75NTR), heart- and neural crest derivatives-expressed 2 (HAND2), cytoglobin, and cysteine and glycine-rich protein 2 (CRP2) have been discussed as HSC specific markers within the liver (Weiskirchen et al., 2001; Suzuki et al., 2008; Iwaisako et al., 2014; Kisseleva 2017; Tsuchida and Friedman, 2017). However, the definition of general markers for HSCs is rather complex because reporter microarray analysis, gene mouse models and single cell RNA sequencing have demonstrated the existence of distinct and functionally relevant subsets of resting HSCs and activated MFBs, both *in vivo* and *in vitro* (Magness et al., 2004; D’Ambrosio et al., 2011; 9,; Krenkel et al., 2019). Nevertheless, the expression of α-SMA and collagen type I is significantly increased during progression of hepatic fibrosis confirming the view that MFBs are still most likely the most relevant cell population contributing to hepatic fibrosis (Figure 7). In addition, the expression of CRP2, Fibulin 2, NGFR, PDGFRβ, Vimentin and many other genes is often used as markers that become increased expressed during hepatic fibrosis (Figure 8).

Recent studies have shown that there exists complex cellular heterogeneity even within activated HSCs that convert into collagen-secreting MFBs. Recent single-cell RNA sequencing (scRNA-seq) studies in a CCl_4_-induced hepatic fibrosis model in mice, clearly showed the presence of four sub-populations of MFBs in the fibrotic liver that all express collagen but differentially express chemokines (Krenkel et al., 2019). Similarly, a very recent human liver scRNA-seq study has revealed HSC heterogeneity along the porto-central axis of the healthy liver lobule (Valery et al., 2021). Two major HSC sub-populations were obtained from the healthy human liver lobules. One sub-population (HSC1) expressed high levels of the cell surface proteoglycan glypican 3 (*GPC3*) and the neurotrophic tyrosine kinase receptor type 2 (*NTRK2*) along with other commonly expressed HSC markers, whereas the second sub-population (HSC2) expressed high levels of the genes encoding for dopamine-norepinephrine converting enzyme (*DBH*), hedgehog-interacting protein (*HHIP*), and the G-protein coupled receptors (GPCRs) vasoactive intestinal peptide receptor 1 (*VIPR1*), parathyroid hormone 1 receptor (*PTH1R*), receptor activity-modifying protein 1 (*RAMP1*), endothelin receptor type B (*EDNRB*), and angiotensin receptor 1A (*AGTR1A*) (Valery et al., 2021). In addition, beside the identification of novel quiescent markers such as Quiescin Q6 sulfhydryl oxidase 1 (*QSOX1*) and six-transmembrane epithelial antigen of prostate 4 (*STEAP4*), these scRNA-seq studies have also confirmed well-established HSC marker including the regulator of G protein signaling (*RGS5*), pleiotrophin (*PTN*), nerve growth factor receptor (*NGFR*), lecithin retinol acyltransferase (*LRAT*), fibulin 5 (*FBLN5*), dihydrolipoamide branched-chain transacylase (*DPT*), decorin (*DCN*), cytoglobin (*CYGB*), collectin 11 (*COLEC11*), olfactomedin-like 3 (*OLFML3*), and tropomysosin 2 (*TPM2*), respectively (Valery et al., 2021). All these findings established by scRNA-seq suggests this methodology as an emerging area and promising experimental tool to reveal deeper insights into HSC biology.

### Portal Fibroblasts

In the normal liver, they encompass a quiescent phenotype with a spindle-shaped fibroblastic phenotype that surrounds the portal vein to maintain integrity of portal tract. In cholestatic liver injury, portal MFBs are supposed to be a more important source of activated MFBs than HSCs around proliferating bile ducts (Tsuchida and Friedman, 2017). However, in contrast to the well-characterized HSCs/MFBs the biology of these cells is only partially known. Studies on rat portal MFB cell lines and fibrotic mouse livers have shown that typical markers of this profibrogenic cells are elastin, type XV collagen α1, ectonucleoside triphosphate diphosphohydrolase-2 (ENTPD2/CD39L1) and cofilin 1, while these cells are negative for the HSC markers desmin, cytoglobin, and LRAT (Iwaisako et al., 2014; Fausther et al., 2015). However, likewise HSCs, these cells are positive for typical myofibroblastic markers including α-SMA, type I collagen α1, and tissue inhibitor of metalloproteinase-1 (Fausther et al., 2015). Other markers for portal MFBs were identified by immunohistochemistry of fibrotic liver and FACS sorting of liver cell preparations. These include Gremlin 1, Thy1/CD90, Fibulin 2, mesothelin, asporin, and Mucin-16 (Iwaisako et al., 2014; Kisseleva, 2017). Some of them are drastically induced during progression of hepatic fibrosis, while their expression signature might dependent on the hepatic insult analyzed (Iwaisako et al., 2014).

### Fibrocytes

Each organ has multiple populations of resident mesenchymal cells capable of producing ECM. In the liver, HSCs and portal fibroblasts are supposed to be the major cell types implicated in the pathogenesis of liver fibrosis. Nevertheless, dependent of the nature of hepatic insult, ECM producing cells may also originate from many other sources. Fibrocytes are defined monocyte-derived spindle-shaped cells having features of both macrophages and fibroblasts (Reilkoff et al., 2011). Animal experimentation using chimeric mice transplanted with donor bone marrow from collagen α1(I)-GFP^+^ reporter mice has shown that collagen-producing fibrocytes are recruited from the bone marrow to the damage liver tissue when recipient mice were subjected to bile duct ligation (Kisseleva et al., 2006). Moreover, when treated in culture with TGF-β1, these cells differentiated into α-SMA and desmin positive collagen-producing MFBs.

### Vascular Smooth Muscle Cells

Vascular smooth muscle cells (VSMCs) are integral components of the blood vessel wall contributing to structural stability and regulating vessel diameter. As such these contractile cells are highly responsive toward vasoactive stimuli and contain a large repertoire of specific contractile proteins facilitating their dynamic phenotype (Metz et al., 2012). In response to injury, VSMCs can shift from a contractile to a synthetic phenotype characterized by increased expression of ECM compounds such as collagen I and III and elevated expression of various non-muscle myosin heavy chain isoforms (Metz et al., 2012). In normal human liver, these cells are positive for α-SMA and smoothelin representing a 59-kD cytoskeletal protein that is found exclusively in contractile smooth muscle cells (Lepreux et al., 2013). During the pathogenesis of advanced human liver fibrosis, the cellular fraction of MFBs positive for both α-SMA and smoothelin expanses to 5–10% suggesting a progressive involvement of these resident cells in MFB recruitment (Lepreux et al., 2013). Comparative transcriptome profiling of endothelial cells and VSMCs from canine vessels revealed an enrichment of expression in genes associated with cytoskeleton composition and actin filament organization including transforming growth factor-β1 (*TGFB1*), collagen type I α1 (*COL1A1*), nephroblastoma overexpressed gene (*NOV*), Tenascin c (TNC), tissue factor pathway inhibitor 2 (*TFPI2*), Tubulin α-4A (*TUBA4A*), Retinol–binding protein (*RBP4*), insulin-like growth factor-binding protein 5 (*IGFBP5*), and Cingulin-like 1 (*CGNL1*) (Oosterhoff et al., 2019). Single cell transcriptomic further showed that VSMC in mouse and human livers can be differentiated from other pro-fibrogenic cells of mesenchymal origin (fibroblasts, HSCs) by their expression of Calponin 1 (*CNN1*) or Myosin heavy chain 11 (*MYH11*) (Dobie et al., 2019).

### Bone Marrow-Derived Fibrocytes

The first hints for a unique population of collagen-producing fibrocytes derived from the bone marrow that could participate in the pathogenesis of hepatic fibrosis were established in chimeric mice transplanted with donor bone marrow from collagen α1(I)-GFP^+^ reporter mice (Kisseleva et al., 2006). In livers of respective mice, a significant increase in GFP^+^/CD45^+^ positive myofibroblastic cells was observed when animals were subjected to bile duct ligation, that however, were not positive for the typical HSC markers α-SMA or vimentin underpinning their lymphoid origin (Kisseleva et al., 2006). However, these cells differentiated into α-SMA and desmin positive cells when cultured in the presence of TGF-β1. A relevant functional contribution of fibrocytes to the pathogenesis of hepatic fibrosis was demonstrated in a mouse model in which fibrocytes were specifically depleted utilizing a herpes simplex thymidine kinase/ganciclovir suicide approach in the thioacetamide-induced liver fibrosis model (Hempel et al., 2019). Although the depletion of fibrocytes resulted in reduced deposition of fibrillar collagen, the antifibrotic effect was not accompanied by a reduction of MFBs. In the multidrug resistance gene 2 knockout (*Mdr2* ^−/−^) mice spontaneously developing cholestatic fibrosis, fibrocytes only minimally contributed to the deposition of ECM in the injured livers (Nishio et al., 2019). It will now be of fundamental interest, to better define the autocrine and paracrine functions of fibrocytes during initiation and progression of hepatic fibrosis in these and other models.

### Hepatocytes

Hepatocytes are specialized epithelial cells making up 80% of the total mass of the liver. They perform numerous vital functions, including protein synthesis, metabolism of lipids and carbohydrates, biotransformation and detoxification of xenobiotics that enter the body. In addition, hepatocytes synthesize and secrete bile and must therefore establish a unique polarity in which apical (canalicular) and basolateral (sinusoidal) plasma membranes are equipped with highly specialized surface proteins, channels, and receptors (Schulze et al., 2019). During liver injury these cells can contribute to fibrogenesis by acquiring myofibroblastic phenotypes/features by undergoing a process termed epithelial-to-mesenchymal transition (EMT) (Zeisberg et al., 2007). During this process the cells downregulate epithelial features, lose their apical-basal polarity, cell-cell adhesion properties and obtain migratory/invasive properties, and acquire mesenchymal characteristics allowing synthesizing ECM compounds (Yang et al., 2020). Lineage-tracing experiments performed in transgenic mice in which liver fibrosis were induced by repeated injections of carbon tetrachloride demonstrated that up to 45% of fibroblast-specific protein 1 (FSP1) positive fibroblasts originated from hepatocytes via EMT (Zeisberg et al., 2007). In line with the concept of EMT, primary mouse hepatocytes transit in culture to FSP1 positive fibroblasts when cultured in the presence of TGF-β1 (Zeisberg et al., 2007). However, the concept that fibrogenic cells capable to express type I collagen can originate *in vivo* from hepatocytes was challenged by other studies (Taura et al., 2010; Xie and Diehl, 2013). It was argued that potential interpretational pitfalls may arise from the fact that FSP1 is not only expressed in subsets of fibroblasts but is also expressed by cells of the myeloid-monocytic lineage (Scholten and Weiskirchen, 2011). However, the evidence for and against EMT for the generation of myofibroblastic cells from intrahepatic cells is still controversially discussed (Taura et al., 2016; Munker et al., 2017; Chen et al., 2020).

### Biliary Epithelial Cells

Similar to hepatocyte it was proposed that biliary epithelial cells (i.e., cholangiocytes) can change their fate and transit to invasive fibroblasts by EMT. In particular, in primary cirrhosis it was demonstrated that bile duct epithelial cells express FSP1 and vimentin as early markers of fibroblasts in the ductular reaction (Robertson et al., 2007). In line, the stimulation of cultured human cholangiocytes with TGF-β induced expression of FSP1 and vimentin suggesting that these cells can contribute significantly to portal tract fibrosis (Rygiel et al., 2008). The resulting cells formed in this localized EMT showed coexpression of both cytokeratin-7 (CK-7) and FSP1 indicating that these cells have the capacity to migrate out of the ductular structure (Rygiel et al., 2008). Several reports suggested that sonic hedgehog signaling promotes EMT by inducing myofibroblast specific genes and repressing epithelial genes during the pathogenesis of chronic biliary injury and NAFLD (Omenetti et al., 2008; Syn et al., 2009). However, bile duct ligation experiments performed in adult mice tagged with a YFP reporter directed under regulatory control of the cholangiocyte marker keratin 19 (K19) showed that cholangiocytes that were positive for YFP revealed no expression of EMT markers α-SMA, desmin, or FSP1 (Scholten et al., 2010).

### Hepatic Progenitor Cells

The liver is the only visceral organ that can replace lost or damaged tissue from the remaining tissue in a well-orchestrated program, in which progenitor cells derived from the biliary epithelium transdifferentiate to restore the hepatocyte compartment (Michalopoulos 2013). Therefore, the occurrence of resident hepatic progenitor cells (HPCs) was proposed that should contain a defined cell fraction located in the canal of Hering. The proposed cells should be characterized by a high cellular plasticity and proliferation potential, the ability to differentiate into hepatocytes and cholangiocytes, and to mediate liver repopulation after injury (Li W et al., 2020). However, also the conversion of hepatocytes to progenitor-like cells has been documented *in vitro* (Li W et al., 2020). HPCs isolated from chronically injured liver were shown to have trilineage differentiation potential serving as progenitors for hepatocytes, cholangiocytes and MFBs Sekiya et al., 2016). Although the frequency of MFBs from HPCs was very low, it can be speculated that HPCs can contribute to the MFB pool during hepatic fibrogenesis (Sekiya et al., 2016).

### Sinusoidal Endothelial Cells

Liver sinusoidal endothelial cells (LSEC) are a fenestrated cell type without an organized basement membrane that forms the predominant population in the hepatic sinusoid. In normal liver, these cells form a selective barrier between the hepatocytes and blood, possess a high endocytotic capacity allowing them to act as an initial line of defense against invading pathogens, and are critically involved in regulating vascular tone and permeability (Hutchins et al., 2013). Under certain conditions these cells can acquire an active phenotype characterized by swelling and bulging of the cell body combined with enlargement of the Golgi complex, increase of rough endoplasmic reticulum, and formation of hemidesmosome-like structures that are hallmarks of fibroblastic reticulum cells (Bardadin and Desmet, 1985). During liver injury LSEC lose their fenestration, form a continuous basal membrane, and develop inflammatory and fibrotic features, a process referred to as capillarization (Baiocchini et al., 2019). Noteworthy, capillarized LSECs can be an active contributor to the production of a fibrotic environment during fibrogenesis by synthesis of collagen and fibronectin (Natarajan et al., 2017).

### Mesothelial Cells

Mesothelial cells are specialized pavement-like cells forming a protective layer of epithelial cells (i.e., the mesothelium) around serous cavities and internal organs. These cells facilitate transport of fluid across these compartments and produce a lubricating fluid that is helpful in protecting the body against infections (Mutsaers 2004). Observations from different animal models and organ systems have shown that the adult mesothelium of mice and humans contains a sub-population of quiescent cells with stem-like properties (Koopmans and Rinkevich, 2018). Upon peritoneal damage and appropriate stimulus, these cells can be triggered to undergo a transition process, termed “mesothelial-to-mesenchymal transition” (MMT). The molecular reprogramming is associated with morphological and functional changes and lead to cells producing ECM compounds and pro-fibrogenic mediators (Koopmans and Rinkevich, 2018). In line, TGF-β1 *in vitro* induced morphologic and functional reformation of differentiated human mesothelial cells to MFBs that become positive for α-SMA (Yang et al., 2003). In regard to liver fibrogenesis, conditional cell lineage tracing in mice confirmed that liver mesothelial cells can be driven by TGF-β to generate both HSCs and MFBs depending on injury signals in the liver (Li et al., 2013). While mesothelial cells preferentially transit into HSCs in biliary fibrosis induced by bile duct ligation, the cells majorly convert into MFBs in carbon tetrachloride-induced fibrosis (Li Y et al., 2016). On the basis of lineage tracing studies, it was supposed that mesothelial cells are triggered by TGF-β to undergo MMT and contribute to the MFB fraction in peritoneal fibrosis, in which up to 16.8% of all MFBs were derived from peritoneal mesothelial cells (Lua et al., 2015).

### GLI1 Positive Perivascular Mesenchymal Stem-like Cells

**FIGURE 9:**
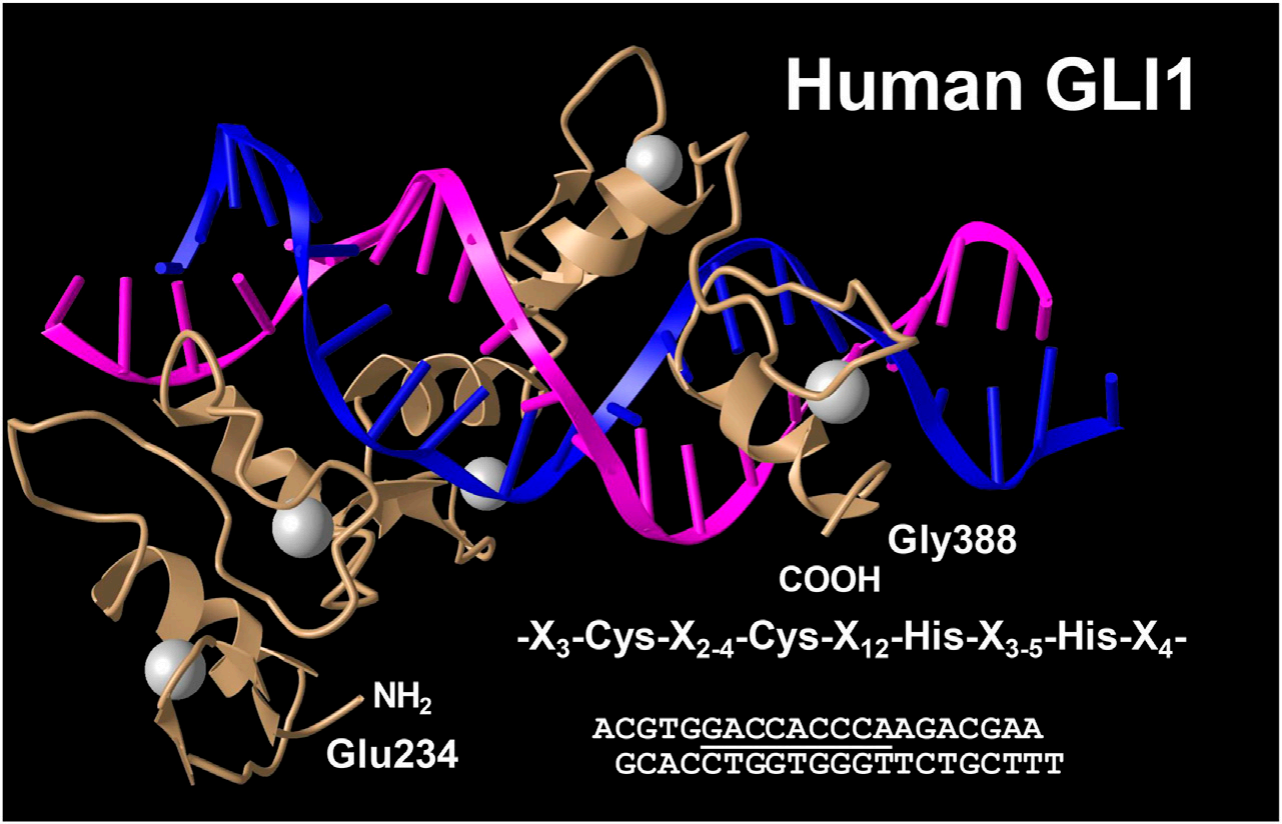
Crystal structure of the five Zn fingers from human GLI1 in complex with a high-affinity DNA binding site. Shown is a complex of a peptide derived from the human GLI1 oncoprotein spanning region Glu 234 to Gly388 with a DNA fragment containing the specific binding site 5′-GACCACCCA-3′ (underlined). Each of the five zinc fingers has a conserved sequence motif that is characterized by the consensus sequence X_3_-Cys-X_2-4_-Cys-X_12_-His-X_3-5_-His-X_4_ (where X is any acid residue). The structure has been determined at 2.6 Å resolution. Structure coordinates were taken from the PDB Protein Data Bank (access. no. 2GLI). For details see (Pavletich and Pabo 1993).

**FIGURE 10:**
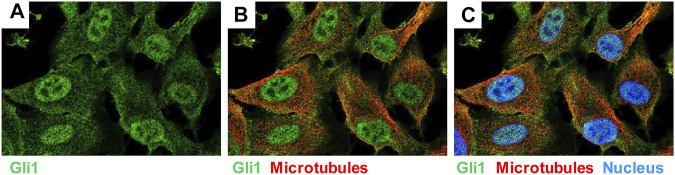
Expression of GLI1 in human bone osteocarcoma cell line U-2 OS. The cell line U-2 OS originating from human mesenchymal tumors express large quantities of GLI1 (*green*), which is localized in the nucleus and the cytoplasm. Microtubuli (*red*) and nucleus (*blue*) are stained by a specific antibody or DAPI. The figure was compiled using immunocytochemical data taken from the Human Protein Atlas v.20 (www.proteinatlas.org/) (Uhlén et al., 2015). They can be found at: https://www.proteinatlas.org/ENSG00000111087-GLI1/cell#img.

**FIGURE 11:**
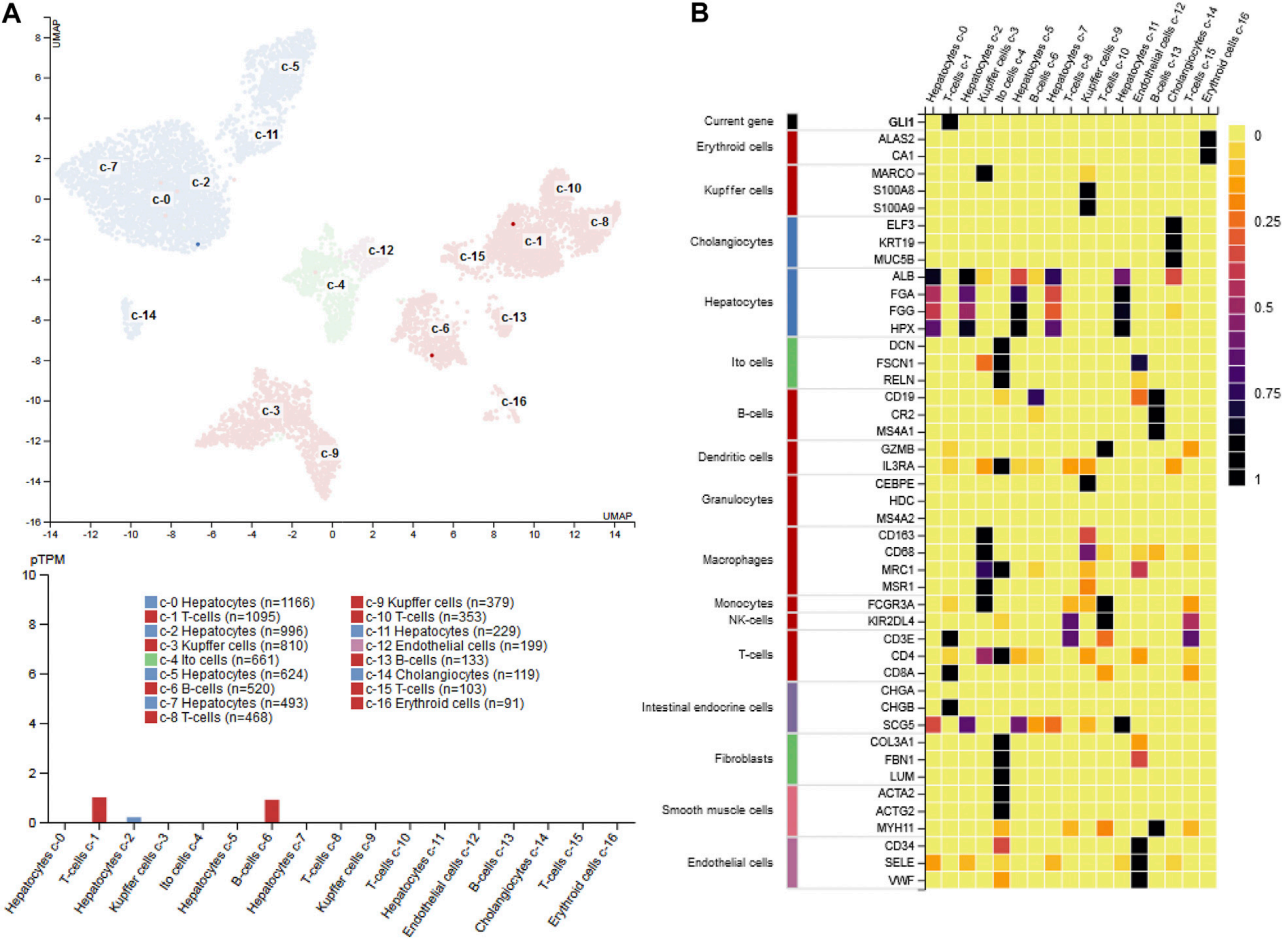
GLI1 expression in liver **(A)** single cell PCR data shows that *GLI1* mRNA expression in normal human liver is rather low (<1 protein-coding transcript per million) and majorly restricted to a subpopulation of T-cells, B-cells and hepatocytes **(B)** Heatmap of marker gene expression in different hepatic cell types. The figure was compiled using expression data from the Human Protein Atlas (www.proteinatlas.org/) (Uhlén et al., 2015). Abbreviations used are: pTPM, protein-coding transcript per million; UMAP, uniform manifold approximation and projection.

The glioma-associated oncogene homolog 1 (GLI1) belongs to the family of three GLI C_2_H_2_-Kruppel type transcription factors that contain five zinc finger domains and either activate or repress gene expression by binding to specific consensus DNA sequences (Figure 9). Traditionally, GLI proteins are viewed as downstream effectors of the Hedgehog (HH) signaling pathways, but are now also known to be regulated transcriptionally and post-transcriptionally through non-canonical mechanisms involving RAS-RAF-MEK-ERK and PI3K-AKT-mTOR (Dusek and Hadden, 2021). This zinc finger protein was originally identified as an oncogene that was amplified more than 50-fold and highly expressed in some cases of malignant glioma (Kinzler et al., 1987). GLI1 localize predominantly to the nucleus (Figure 10) and bind the 9-base-pair consensus DNA 5′-GACCACCCA-3′ with high affinity (Kinzler and Vogelstein, 1990). It has turned out that the individual GLI proteins play fundamental and distinct roles both in chronic inflammation and cancer. In some organs the lack of HH expression promotes chronic inflammation and tumor formation, while aberrantly activated HH/GLI signaling is also capable to foster tumor growth and simultaneously dampening inflammation and favoring immunosuppression (Grund-Gröschke et al., 2019). Genetic lineage tracing analysis in mice demonstrated that tissue-resident, but not circulating, GLI1 positive mesenchymal-stem-cell-like cells can generate MFBs in kidney, lung, liver, or heart after injury (Kramann et al., 2015). Genetic ablation of GLI1 positive cells abolished bone marrow fibrosis and rescued bone marrow failure (Schneider et al., 2017). More recently it was demonstrated that the pro-fibrogenic activity of osteopontin in promoting HSC activation and ECM deposition during liver fibrogenesis is strongly dependent on GLI1 function (Rao et al., 2019). In the human HSC line LX-2, PAX6 binds to the promoter of the *GLI1* gene, thereby promoting fibrogenic activities and proliferation (Li C. et al., 2020). In the same cell line, GLI1 was further shown to be integrated in a complex network of Wnt/β-catenin, which regulates cellular contraction (Zhang F. et al., 2020). However, the significance of GLI1 positive perivascular mesenchymal stem-like cells for liver fibrogenesis is still unknown. Publicly available data obtained by single cell PCR shows that *GLI1* mRNA expression in normal human liver is rather low (<1 protein-coding transcript per million) and restricted to some immune cells and hepatocytes (Figure 11), while not found in smooth muscle cells or endothelial cells. It will be now of particular interest to document the existence of respective cells and to clarify how these cells are triggered during hepatic fibrogenesis to generate the proposed large fraction of MFBs.

## Mechanisms of Fibrosis Regression and Resolution

Liver fibrosis is potentially reversible (Ramachandran and Iredale, 2012). Patients undergoing treatment for HCV infection clearly demonstrate reversal of fibrosis upon complete HCV negativity (Brenner, 2013). However, liver fibrosis is reversible only in the early stages (Fibrosis grades 1 and 2). Once fibrosis crosses a threshold (Fibrosis grades 3 and 4), the fibrogenic type I collagen forms crosslinks and is typically associated with cell damage and inflammation, making it harder to recover (Brenner, 2013). Two events are critical in directing the liver pro-fibrogenic phenotype to recovery - (i) Apoptosis of MFBs in the liver and, (ii) switching of macrophages from a pro-inflammatory to a tissue resolution phenotype (Pellicoro et al., 2014). During liver fibrogenesis, the ECM is extensively remodeled leading to accumulation of proteases such as matrix metalloproteinases (MMP) as well as collagenases (Iredale, 2008). However, at the same time, fibrotic liver also accumulates myofibroblast-derived tissue inhibitor of metalloproteinase 1 (TIMP1) which prevents the action of MMPs and ECM turnover (Iredale et al., 1996). As a result, there is an accumulation of collagen and pro-fibrotic ECM. Over a period of time, accumulation of a large number of crosslinked collagen and elastin fibers lead to sequestering of crosslinked fibers within the tissue beds, making them inaccessible for proteolytic digestion (Issa et al., 2004). As the cross links increase, the exposed fibers also become less susceptible to digestion themselves (Issa et al., 2004). The hallmark of a recovering fibrotic liver is the termination of cellular injury followed by the absence or disappearance of hepatic MFBs (Kisseleva et al., 2012). Studies show that at least 50% of the activated MFBs revert to less fibrogenic or quiescent HSCs (Kisseleva et al., 2012; Troeger et al., 2012). The role of macrophages and the trigger of switching from pro-inflammatory to pro-resolution macrophages during fibrosis is incompletely understood. However, macrophages in the resolving fibrotic liver have been shown to secrete increased levels of MMPs thereby contributing to ECM reorganization (Li H et al., 2016). Therefore, polarization of macrophages provides a therapeutic opportunity for the resolution of liver fibrosis.

## Therapy of Hepatic Fibrosis

**FIGURE 12:**
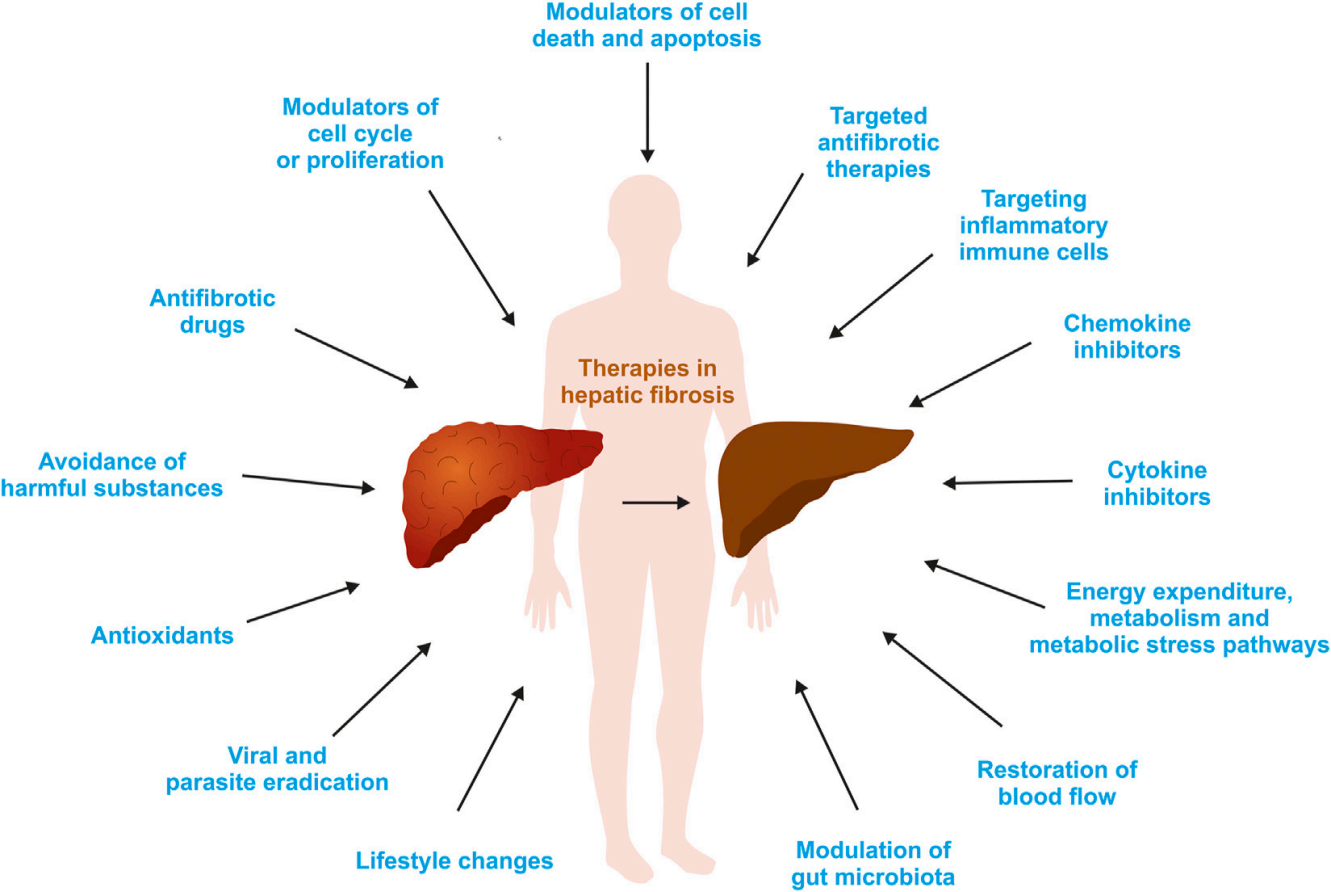
Potential therapeutic options for liver fibrosis. Based on the fact that hepatic fibrosis is driven by different mediators and pathways, there is a plenitude of possibilities to interfere with this process. For more details see text or refer to (Schon et al., 2016; Weiskirchen 2016; Tacke and Weiskirchen, 2018; Weiskirchen et al., 2018; Levada et al., 2019).

Although numerous drugs have beneficial anti-fibrotic effects *in vitro* and in animal models, none of these drugs has been ultimately shown to be efficacious in the clinic. Moreover, general anti-fibrotic therapies are not available. Instead, clinicians and professional associations have developed some clinical practice guidelines and recommendations for etiology-specific interventions. Most noticed are the guidelines published by the American Association for the Study of Liver Diseases (AASLD) and the European Association for the Study of Liver Diseases (EASL) that both develop evidence-based clinical practice guidelines on a regularly basis. These ‘state-of-the-art’ recommendations are intended to assist physicians and other healthcare providers in the diagnosis and management of a specific etiology of liver injury. As such they typically contain information about disease definition, epidemiology, etiology, risk factors, incidence, recommended tests and examinations for disease detection, screening tools, preferred staging and grading systems, therapy strategies, surveillance tests/intervals, therapy outcome measures, prevention strategies, ongoing trials, and much other supporting information. From the view of basic scientists some generally applicable concepts should be effective in the therapy of hepatic fibrosis. These include the withdrawal of injurious stimuli, inhibition of ongoing hepatic damage, deactivation and elimination of ECM-producing cells, removal of superfluous scar tissue, counteracting biological mediators driving hepatic inflammation and fibrogenesis, and restoring the normal liver architecture (Figure 12). scRNA-seq and genetic cell tracing experiments have shown that the termination of hepatic fibrosis is associated with a reversal of HSC activation and expression of different inactivation markers (Troeger et al., 2012). However, reverted HSCs remain in a primed state maintaining a higher responsiveness toward fibrogenic stimuli.

### Arrest of Chronic Liver Damage by Avoiding or Eradication of Harmful Substances and Replacement Therapies

As outlined, there are many genetic and environmental factors that can cause hepatic fibrosis. Most frequent inherited disorders associated with acute and chronic liver disease include hemochromatosis, Wilson disease, α1-antitrypsin deficiency, and cystic fibrosis. In the case of hemochromatosis, excessive iron can be removed from the body by regularly phlebotomy, or alternatively iron chelating therapy (Murphree et al., 2020). Wilson disease occurring as a consequence of impaired biliary copper excretion can be effectively treated with the chelators d-Penicillamine and trientine or by application of zinc preparations interfering with the gastrointestinal uptake of copper (Stremmel and Weiskirchen, 2021). Shortage of α1-antitrypsin in the lung can be partially overcome by intravenous replacement therapy, while this therapy is not appropriate to people with liver disease. In respective patients, there is emphasis on efforts to prevent progression of related liver injury by reducing of modifiable risk factors (overweight, tobacco, alcohol, non-steroidal anti-inflammatory drugs) or finally liver transplantation that still remains the sole curative option (Narayanan and Mistry, 2020). In cystic fibrosis resulting of mutations in the gene encoding the cystic fibrosis transmembrane conductance regulator (CFTR) the application of Ursodeoxycholic acid (UDCA) is the mainstay of therapy. This secondary bile acid is supposed to stimulate bile acid secretion, but its efficacy is therapy of cystic fibrosis-related liver disease is controversially discussed (Staufer, 2020). In addition, a large number of compounds restoring CFTR protein function (i.e., CFTR modulators) are actually under close investigation (Staufer, 2020).

In regard to alcoholic liver disease, the abstinence from drinking alcoholic beverages is quite the cornerstone of therapy. However, several studies have shown that glucocorticoids given alone or in combinations with antioxidants are beneficial to lower hepatic inflammation (Mitchell et al., 2020). In addition, drugs able to prevent the development of steatosis, modulate innate immune responses, targeting the microbiome, or stimulating liver regeneration are investigated in many clinical studies (Mitchell et al., 2020).

Antiviral treatment strategies are suitable to reduce the burden of chronic hepatitis B (HBV), hepatitis C virus (HCV), or hepatitis D (HDV) infections. In particular, the introduction of direct antiviral drugs offers nowadays a very competent way to obtain viral clearance with sustained virologic response rates greater than 95% (Do and Reau, 2020). Rigorous infant prophylaxis, early childhood and adult immunization programs as well as vaccination of high-risk individuals significantly contribute to prevalence of HBV transmission worldwide (Polaris Observatory Collaborators, 2018). Established interferon-based therapies are established and new encouraging drugs are currently under clinical evaluation for the treatment of HDV (Koh et al., 2019).

Autoimmune diseases of the liver typically affect either the liver parenchyma, which is termed autoimmune hepatitis, or alternatively the bile ducts provoking primary biliary cholangitis (PBC) or primary sclerosing cholangitis (PSC) (Weiskirchen, 2016). Immunosuppressive treatment consisting of either corticosteroids alone or in combination with the purine analog azathioprine is the recommended first-line medical treatment in autoimmune hepatitis (Tanaka, 2020).

There are some pharmacological approaches available for the management of NAFLD and NASH, but an ultimate therapy is still missing and actual guidelines presently only recommend significant changes in lifestyle and nutrition, in particular weight loss and physical exercise (Drescher et al., 2019). Nevertheless, there are several drugs currently at various stages of development for the therapy of NASH, possessing anti-inflammatory activity, improve insulin resistance, reduce *de novo* lipogenesis, modulate lipid transport or oxidation, or evolve anti-apoptotic effects (Tacke and Weiskirchen, 2018).

Liver fibrosis resulting from Schistosomiasis (*S. mansoni* and *S. japonicum*) are presently treated with the pyrazinoisoquinoline derivative praziquantel, while several vaccines that are urgently needed are currently at differing phases of clinical development and not yet been accepted for public use (McManus et al., 2020).

### Antioxidants

As discussed, elevated quantities of reactive oxygen species (ROS) are key drivers of hepatic inflammation and fibrosis. Under normal condition, ROS are required for many important signaling processes, impact cell proliferation, contribute to apoptotic pathways, and help phagocytic active cells to destroy and eliminate pathogens (Luangmonkong et al., 2018). They induce apoptosis and necrosis of parenchymal cells (i.e., hepatocytes) resulting in the release of harmful mediators (e.g., TGF-β, TNF-α), stimulate Kupffer cells to produce profibrogenic mediators, prompt recruitment of circulating inflammatory cells into the liver, and contribute to the activation of HSCs (Weiskirchen, 2016). Therefore, an imbalance between ROS production and degradation play an important role in the pathogenesis of liver fibrosis. Consequently, therapeutic interventions targeting elevated cellular oxidative stress should be beneficial for the treatment of liver fibrosis (Luangmonkong et al., 2018). In regard to therapy of liver fibrosis, many ROS inhibitors have been tested successfully in pre-clinical animal models (Weiskirchen, 2016). Most of these antioxidants are scavengers that unspecific alleviate ROS accumulation, while others are more selective by targeting defined molecular pathways involved in ROS generation. In particular, inhibitors of mitochondrial dysfunction (Coenenzyme !0, Mitoquinone mesylate, NIM811), endoplasmic stress (Glycerol phylbutyrate), NADPH oxidases (GKT137831, Docosahexaenoic acid, losartan), and Toll-like receptors (Curcumin, Quercetin, various probiotics, Bicyclol) have attracted widespread attention in recent years (Luangmonkong et al., 2018). Most promising are drugs that interfere with the activity of different NADPH oxidase (NOX) subtypes. In experimental models, both the deficiency of NOX1 or NOX4 as well as the application of the dual NOX1/4 inhibitor GKT137831 was effective in attenuation of carbon tetrachloride-induced liver fibrosis (Lan et al., 2015). Likewise, the NOX inhibitor apocynin was therapeutically effective in preventing lipopolysaccharide/d-galactosamine-induced acute liver injury (Peng et al., 2020).

### Inhibitors of Cytokine Signaling

Several cytokines play a crucial role in the pathogenesis of hepatic fibrosis. Commonly, they bind to specific cell-surface exposed receptors, thereby initiating intracellular signaling cascades ending in modified gene expression. Research performed during the last decades has identified a number of different cytokines relevant during the pathogenesis of hepatic fibrosis. Representative cytokines most prominent involved in disease initiation or progression are members belonging to the family of TGF-β, platelet-derived growth factors (PDGF), vascular endothelial growth factors (VEGF), interleukins (IL), fibroblast growth factors (FGF), interferons (IFN), insulin-like growth factors (IGF), TNF-α, epidermal growth factor (EGF), nerve growth factor (NGF), and hepatocyte growth factor (HGF) (Weiskirchen 2016). Their specific activities were comprehensively explored in many *in vitro* and *in vivo* models of hepatic fibrosis. However, proposed therapies by interfering with cytokine activities using small interfering RNAs, antisense oligonucleotides, aptamers, soluble receptors, scavenger molecules, therapeutic antibodies, or other biological agents have not been translated to the clinics yet (Borkham-Kamphorst and Weiskirchen, 2016; Weiskirchen, 2016; Schuppan et al., 2018).

### Inhibitions of Chemokine Signaling

Chemokines are critical immunomodulatory mediators acting in humans through 20 different G-protein-coupled transmembrane receptors. They typically consists of 75–125 amino acids, share a similar tertiary structure, and based on the number and position of cysteine residues can be systematically categorized into four distinct subfamilies, namely CC, CXC, CX_3_C, and XC followed by the letter L (standing for ‘ligand’) and a consecutive number indicating their temporal isolation (Hughes and Nibbs, 2018). The individual ligands and their cognate receptors (i.e., CCR, CXCR, CX_3_CR, XCR) form an enormously complex network playing pivotal roles. By far the most studied functions are the control of cell recruitment, inflammation, wound healing, lymphoid trafficking, angiogenesis, and metastasis. For the formation of liver disease, there is now ample evidence that chemokines and their receptors have fundamental importance in both progression and regression of hepatic fibrosis (Marra and Tacke, 2014). Therefore, strategies for inhibiting common or individual chemokine activities are presently intensively investigated. Prototypically, the dual specific CC motif chemokine receptor 2/5 (CCR2/CCR5) antagonist cenicriviroc has been experimentally and clinically shown to block fat accumulation, Kupffer cell activation, monocyte recruitment, HSC activation, and fibrosis (Friedman et al., 2018; Krenkel et al., 2018; Ambade et al., 2019).

### Other Anti-fibrotic Therapy Strategies

Besides the elimination of pathogenic causes, usage of replacement therapies, usage of antioxidants, or therapies targeting cytokine or chemokine activities, there are numerous other possibilities to interfere with hepatic fibrosis. In the past, many other drugs or herbal supplements or vitamins were experimentally tested in pre-clinical models of hepatic fibrosis (Weiskirchen, 2016). They act by inducing apoptosis, autophagy or senescence in ECM-producing cells, interfere with pro-fibrogenic target molecules, modulate cell cycle or proliferation synthesis, act hepatoprotective, or generally interfere with gene expression, replication, mitosis or meiosis. However, their efficacy was only successfully proven yet in experimental disease models. Clinical application is hindered in most cases because effective strategies that allow targeting these drugs to fibrogenic effector cells are not available (Schuppan et al., 2018). Other experimental approaches have identified the lysyl oxidase-like 2 (LOXL2) encoding an extracellular copper-dependent amine oxidase catalyzing the covalent cross-linking of collagen and elastin as a promising drug target (Khurana et al., 2021). In two experimental models of hepatic fibrogenesis, the selective LOXL2/3 inhibitor PXS-5153A was shown to dose-dependently diminish collagen content, thereby reducing disease severity and improve liver function (Schilter et al., 2019). In line, the preventive treatment with and anti-LOXL2 antibody was able to prevent ongoing experimental hepatic fibrosis (Ikenaga et al., 2017). Similarly, targeting Galectin-3 representing a 30 kDa protein with important functions in cell-cell adhesion, cell-matrix interaction, angiogenesis, macrophage activation, inflammation, and collagen synthesis has been identified as a suitable drug candidate.

Presently, there is much hope that engineered nanoparticles, magnetic-assisted drug delivery techniques, or therapeutic effective transgenes expressed under fibrosis-related promoters can be optimized in the near future to better target individual fibrogenic cell subpopulations (Herrmann et al., 2004; Schon et al., 2016; Levada et al., 2019).

## Conclusion

Genetic disorders, alcohol abuse, drugs, cholestasis, metabolic disorders, chronic viral hepatitis, parasitic infections and several cryptogenic causes are major causes that provoke liver fibrosis. During this progressive process accumulation of ECM, disruption of the lobular structure, and progressive deterioration of hepatocellular function lead to fatal complications. In particular, the exuberant collagen deposition is one hallmark of fibrogenesis. Work from the last decades have identified a number of different resident and infiltrating cells that can either be activated or transform into a phenotype capable to synthesize ECM. In addition, cell- and animal-based experiments, clinical studies and complex integrated bioinformatics analysis have unraveled soluble mediators, molecular pathways, and pro-fibrogenic genes that are key drivers in the pathogenesis of hepatic disease. However, despite the important progress, there are currently no approved anti-fibrotic drugs that have been ultimately shown to be efficacious in the clinic. Presently, clinical practice guidelines are only etiology-specific. They intend to optimize patient care by withdrawal of injurious stimuli, consumption of antioxidant acting compounds, and lifestyle interventions including healthy food, exercised and controlled weight loss. Nevertheless, many experimental studies and clinical trials are currently being conducted to test drugs targeting more specifically inflammation, the cellular activation process, or the activity of inflammatory or fibrotic-acting cytokines or chemokines. There is hope that these compounds will be of fundamental importance in future treatments aiming at impeding or reversing the fibrogenic process.
